# Kinetic Studies on the 2-Oxoglutarate/Fe(II)-Dependent Nucleic Acid Modifying Enzymes from the AlkB and TET Families

**DOI:** 10.3390/dna3020005

**Published:** 2023-03-30

**Authors:** Zhiyuan Peng, Jian Ma, Christo Z. Christov, Tatyana Karabencheva-Christova, Nicolai Lehnert, Deyu Li

**Affiliations:** 1 Department of Biomedical and Pharmaceutical Sciences, College of Pharmacy, University of Rhode Island, Kingston, RI 02881, USA; 2 Department of Chemistry, Michigan Technological University, Houghton, MI 49931, USA; 3 Department of Chemistry and Department of Biophysics, University of Michigan, Ann Arbor, MI 48109, USA

**Keywords:** kinetics, 2-OG-dependent enzyme, AlkB, ALKBH protein, FTO, TET

## Abstract

Nucleic acid methylations are important genetic and epigenetic biomarkers. The formation and removal of these markers is related to either methylation or demethylation. In this review, we focus on the demethylation or oxidative modification that is mediated by the 2-oxoglutarate (2-OG)/Fe(II)-dependent AlkB/TET family enzymes. In the catalytic process, most enzymes oxidize 2-OG to succinate, in the meantime oxidizing methyl to hydroxymethyl, leaving formaldehyde and generating demethylated base. The AlkB enzyme from *Escherichia coli* has nine human homologs (ALKBH1–8 and FTO) and the TET family includes three members, TET1 to 3. Among them, some enzymes have been carefully studied, but for certain enzymes, few studies have been carried out. This review focuses on the kinetic properties of those 2-OG/Fe(II)-dependent enzymes and their alkyl substrates. We also provide some discussions on the future directions of this field.

## 1. Introduction

**Figure 1.**
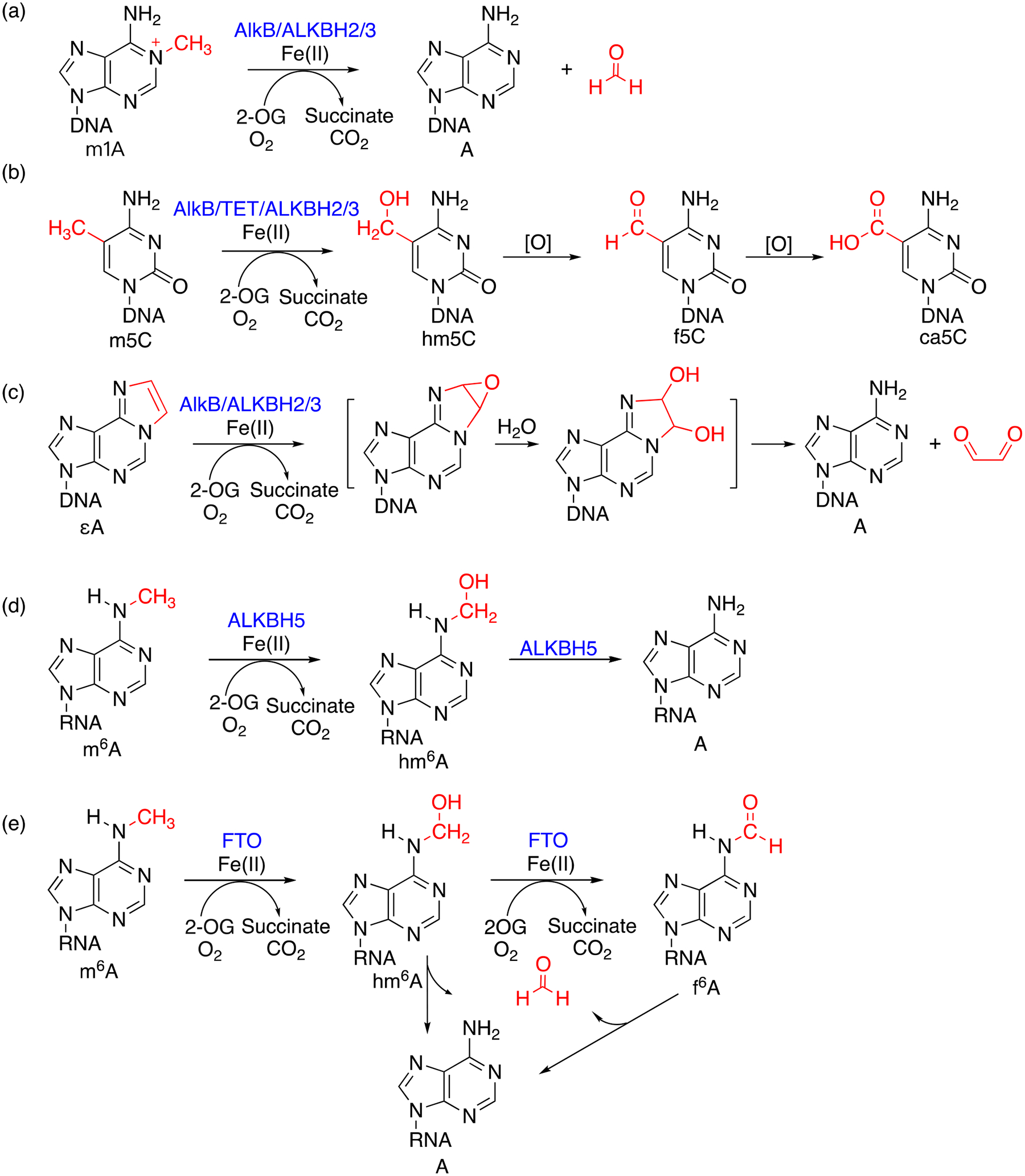
Proposed mechanisms of oxidative modifications on representative substrates catalyzed by 2-OG/Fe(II)-dependent dioxygenases. (**a**) m1A repaired by AlkB, ALKBH2 and 3; (**b**) m5C oxidized by AlkB, ALKBH2 and 3 and TET; (**c**) εA repaired by AlkB, ALKBH2 and 3; (**d**) m6A demethylated by ALKBH5; and (**e**) m6A demethylated by FTO.

**Figure 2.**
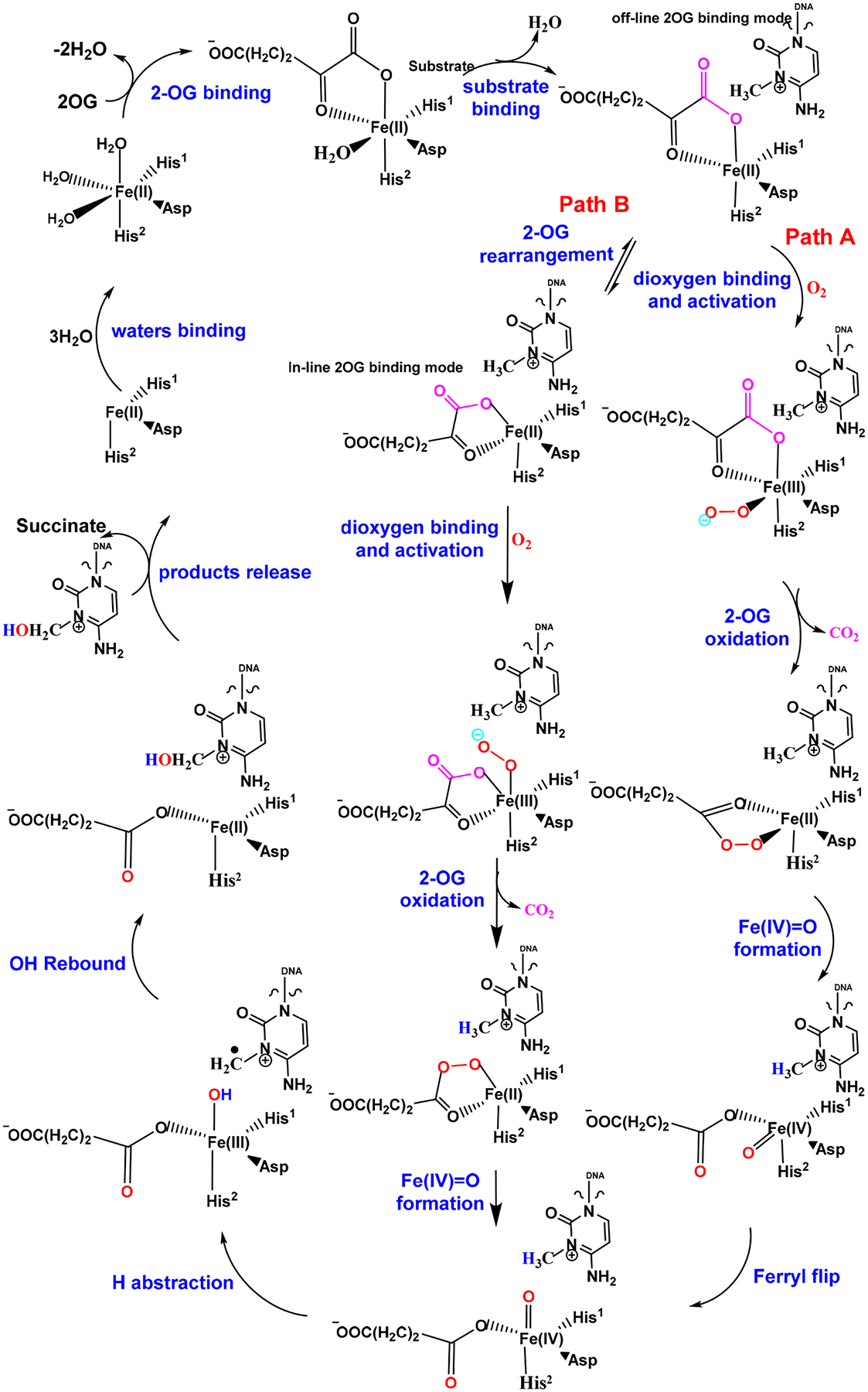
Proposed detailed mechanism of AlkB/TET family enzymes on monoalkyl substrates (exemplified with m3C). The steps include 2-OG binding, DNA/RNA substrate binding, dioxygen binding and activation, 2-OG oxidation, Fe(IV)=O formation, ferryl flip, H abstraction, OH rebound, product release, water binding, etc. Adapted from Scheme 1 in [20].

DNA and RNA are modified by exogenous and endogenous chemicals, such as methyl methanesulfonate (MMS) [1], dimethyl sulfate [2], acrolein, malondialdehyde [3], S-adenosylmethionine (SAM), and PUFA (polyunsaturated fatty acids), causing a variety of modifications [4–6]. *Escherichia coli* AlkB protein is one of the four proteins (Ada, AlkA, AlkB, and AidB) induced during the adaptive response to counteract the attack of alkylating agents [7–10]. AlkB was subsequently discovered as an Fe(II)/2-oxoglutarate (or α-ketoglutarate, 2-OG or α-KG)-dependent dioxygenase (Figure 1) [11–14]. This dioxygenase uses both oxygen atoms from O_2_ during the repair; one oxygen is utilized in the hydroxylated nucleic acid product and the other is used for the oxidation of the co-substrate 2-OG to succinate [15–17]. 2-OG-dependent dioxygenase was first discovered in 1967: Hutton et al. reported an oxygenase-dependent reaction catalyzed by collagen prolyl hydroxylase (CPH) that requires 2-OG [18]. Since this discovery, scientists have identified many 2-OG-dependent enzymes in the biological processes of plants and animals [19]. The TET/JBP family also belongs to the 2-OG oxygenases. The AlkB, TET and JBP family enzymes have been studied extensively for their DNA/RNA modification activities. The overall reaction mechanism of the AlkB/TET enzymes follows the general strategy of non-heme Fe(II)/2-OG enzymes that includes 2-OG binding and DNA/RNA substrate binding, followed by dioxygen binding and activation, hydrogen atom transfer, rebound hydroxylation and product release (Figure 2) [20].

Initially, AlkB was reported to oxidize 1-methyladenine (m1A) and 3-methylcytosine (m3C) in DNA, with loss of formaldehyde and recovery of the unmodified bases [13,14]. Later, this enzyme was found to repair lesions in RNA as well [11]. Other substrates include 1-methylguanine (m1G), 3-methylthymine (m3T) [21,22], *N*^2^-methylguanine (m2G) and *N*^4^-methylcytosine (m4C) [23], *N*^6^-methyladenine (m6A) [24], 1,*N*^6^-ethanoadenine (EA) [24], 1,*N*^6^-ethenoadenine (εA) [25,26], and other adducts [27,28]. AlkB human homologs have been identified as ALKBH1–8 [29] and FTO [30] (also referred as ALKBH9). ALKBH2 and ALKBH3 have been reported to repair m1A and m3C in DNA [31], and ALKBH3 can also repair lesions in RNA [11]. Later, other homologs were investigated for their dealkylation activities. For example, FTO (fat mass and obesity associated [32]) was discovered to demethylate m6A in DNA or RNA (Figure 1) [33]. TET family enzymes (TET1–3) oxidize 5-methylcytosine (m5C) in DNA in successive steps to 5-hydroxymethylcytosine (hm5C), 5-formylcytosine (f5C), and 5-carboxylcytosine (ca5C) (Figure 1) [34–40]. JBP family enzymes (JBP1 and 2) can perform the oxidative hydroxylation of thymine to 5-hydroxymethyluracil (hm5U) in the biosynthesis of Base J (β-D-glucosyl-hydroxymethyluracil) [41–43]. For recent progresses on the AlkB, TET and JBP family enzymes, please see several review articles [44–47]. In order to determine whether a certain substrate is either a strong or weak substrate of a protein, kinetic study is a reliable way to distinguish it. This review mainly focuses on the kinetic behaviors of these enzymes in reactions with different DNA/RNA substrates. Some other studies aimed to investigate the enzyme–substrate complex formation and individual steps of the reaction pathways [48–50].

Non-enzymatic methylations from endogenous SAM [6] or exogenous methylating agents, such as MMS [1] and dimethyl sulfate [2], are the major sources that generate methylated modifications. Some modifications, including m3C, m1G, and m3T, are mutagenic [21], while others, including m6A, m4C and m2G in DNA, do not disrupt Watson–Crick base pairing and thus are not mutagenic [23]. Epigenetic marker m5C constitutes 60–80% of human genomic DNA on the CpG islands [51,52]; it also appears on non-CpG methylation [53]. m6A is 0.1–0.4% of total adenosine residues in cellular RNA [54]. Etheno-DNA lesions are a type of highly mutagenic and toxic biomarker; they are formed from products of either lipid peroxidation (LPO) or the carcinogen vinyl chloride and its derivatives [55]. Several etheno-DNA biomarkers, including εA, 3,*N*^4^-ethenocytosine (εC), 3,*N*^4^-etheno-5-methylcytosine (ε5mC), 1,*N*^2^-ethenoguanine (1,*N*^2^-εG), and *N*^2^,3-ethenoguanine (*N*^2^,3-εG), have been characterized [56,57]. Until now, there are still many enzymes (ALKBH 4, 6, 7, 8, TET 1 and 3, and JBP1 and 2) that have not been kinetically investigated. This review aims to provide insights into the kinetic behavior of the 2-OG/Fe(II) enzymes and offers discussions on the methods of those studies. The enzymes and their substrates are summarized in Table 1, and the kinetic parameters are summarized in Tables 2–4.

## 2. Kinetic Studies of the AlkB and TET Family Enzymes

### 2.1. ALKBH1

Initially, ALKBH1 was the first mammalian AlkB homolog identified in 1996 [83]. ALKBH1 shows strong homology with AlkB (23% identity and 59% similarity) [11,84]. ALKBH1 was reported as a histone dioxygenase that modifies histone H2A methylation status [85]. ALKBH1 has also been reported to repair multiple DNA/RNA substrates: it can demethylate m3C in both DNA/RNA [86,87] and m5C in tRNA [88,89]. Additionally, ALKBH1 is involved in the demethylation of m6A in genomic DNA [58,90] and demethylation of m1A within cytoplasmic tRNAs [91].

To the best of our knowledge, only ALKBH1-mediated oxidation of m6A and m1A have been reported with kinetic data (Table 2). It was found that ALKBH1 demethylates m6A in the single-stranded regions of the mammalian genome. First, enzymatic profiling studies have determined that ALKBH1 prefers bubbled or bulged DNAs as substrates, instead of single stranded (ss)-DNA or double stranded (ds)-DNA. Additionally, enzymatic kinetic analyses were carried out with bulged DNA (k_cat_/K_m_ = 0.043 min^−1^ μM^−1^) compared to ss-DNA (k_cat_/K_m_ = 0.027 min^−1^ μM^−1^) to support these findings. Kinetic studies of m1A repair by ALKBH1 were also performed [91]. In the report, since ALKBH1 has a tRNA binding motif, the authors measured the maximal velocity values of m1A demethylation toward the stem-loop probes to mimic the TΨC loops of tRNA, which have a much higher rate than that towards the unstructured probes. This implies that ALKBH1 has a high preference for the stem-loop structure. The authors performed steady-state kinetics of the ALKBH1-catalyzed demethylation of m1A in the stem-loop structured RNA and unstructured RNA probes. Both ALKBH1 kinetic and other substrate studies indicate that ALKBH1 may prefer bulged DNA or stem-loops of tRNAs over ss- and ds-DNA/RNA.

### 2.2. ALKBH2

ALKBH2 shares the most similar substrate preference with AlkB and is classified as a bona fide DNA repair enzyme together with ALKBH3. ALKBH2 performs demethylation more efficiently on ds-DNA than ss-DNA, while ALKBH3, also an active DNA/RNA demethylase, prefers ss-DNA [11]. Structure analysis of ALKBH2 shows that it uses a finger residue to search for and flip the damaged base into the active site; this specific binding mode ensures the lesion is repaired [92]. The divergent F1 β-hairpins in the vicinity of the active sites of ALKBH2 and ALKBH3 are important for their selectivity: after switching the F1 sites between both proteins, their strand preference also switched [93]. Similar results were obtained for swapping the ss- or ds-DNA preference of ALKBH2 and ALKBH3 by changing the relevant binding motifs [94].

Major substrates for ALKBH2 are m3C and m1A [11,31], together with other minor substrates, such as m3T [22] and m1G [95]. m5C can be oxidized by ALKBH2 [96]. Exocyclic etheno lesions εA, εC, 1,*N*^2^-εG [56] are also repaired by ALKBH2. ALKBH2 is reported to repair 3-ethylthymine [97] and 1-ethyladenine [31]. Since m1A and m3C DNA lesions exist in our body and ALKBH2 repairs them most effectively among all the substrates, ALKBH2 repair of these two substrates has been intensively studied for the kinetic behavior of these reactions. The reaction mechanism of AlkBH2 has also been studied computationally using QM/MM and MD methods [20]. Importantly, these studies revealed an influence of the ds-DNA on flexibility, enzyme dynamics and long-range correlated motions in the reaction pathway.

#### 1-Methyladenine.

Because 2-OG, nucleic acid modification, and molecular oxygen are the cosubstrates of the 2-OG/Fe(II)-dependent reactions, every one of them could be used as a variable substrate for a kinetic study. The kinetic studies of m1A were completed using either DNA or 2-OG as substrates (Table 2) [61]. The results with 2-OG as co-substrate demonstrate that ALKBH2 repairs m1A in ds-DNA (k_cat_/K_m_ = 0.34 min^−1^ μM^−1^) faster than ss-m1A (k_cat_/K_m_ = 0.27 min^−1^ μM^−1^). Similarly, when using DNA as substrate, ALKBH2 can repair m1A in ds-DNA (k_cat_/K_m_ = 0.74 min^−1^ μM^−1^) more efficiently than ss-m1A (k_cat_/K_m_ = 0.25 min^−1^ μM^−1^) [61]. Furthermore, ALKBH2 is at least twice efficient at removing m1A and m3C from ds-DNA [m1A (k_cat_/K_m_ = 2572.5 min^−1^ μM^−1^) and m3C (k_cat_/K_m_ = 3176.0 min^−1^ μM^−1^)] compared to single-stranded DNA [m1A (k_cat_/K_m_ = 1082.0 min^−1^ μM^−1^) and m3C (k_cat_/K_m_ = 773.7 min^−1^ μM^−1^)]. Kinetic studies were also performed on a novel methylation-sensitive nucleic acid (RNA) probe of m1A [CGCGm1AAUUCGCG (k_cat_/K_m_ = 3.4 min^−1^ μM^−1^)] [60], which switches conformation according to its methylation status. Combined with differential scanning fluorimetry measurements, this enables highly sensitive and selective detection of demethylase activity at a single methylated base level. As a result of the CGCGm1AAUUCGCG self-complementary nature, it can inherently adopt a bimolecular duplex through intermolecular base pairing and a monomolecular hairpin by intramolecular pairing. ALKBH2 kinetic properties on regular sequences [ss-RNA (k_cat_/K_m_ = 4.30 min^−1^ μM^−1^) and ds-RNA (k_cat_/K_m_ = 7.4 min^−1^ μM^−1^)] were also reported [60].

#### 3-Methylcytosine.

For the previously mentioned m1A kinetic studies, similar experiments were also performed on m3C [61]. The kinetic parameter for ALKBH2 repairing m3C with 2-OG as co-substrate is k_cat_/K_m_ = 1.3 min^−1^ μM^−1^ for ds-DNA, which is also better than the repair of ss-m3C (k_cat_/K_m_ = 1.2 min^−1^ μM^−1^). When DNA is used as substrate, ALKBH2 repairing m3C also prefers m3C in ds-DNA (k_cat_/K_m_ = 1.10 min^−1^ μM^−1^) over ss-m3C (k_cat_/K_m_ = 0.53 min^−1^ μM^−1^) Lee et al. [61] reported that ALKBH2 is much more efficient at removing m3C in ds-DNA (k_cat_/K_m_ = 3176.0 min^−1^ μM^−1^) compared to ss-DNA (k_cat_/K_m_ = 773.7 min^−1^ μM^−1^ [59]. Theoretical studies delineated the reaction mechanism of AlkBH2 with ss-DNA and ds-DNA containing m3C and revealed the key interactions involved in the catalysis [20].

### 2.3. ALKBH3

ALKBH3, another well-studied AlkB homolog, has been found to actively demethylate m1A and m3C in DNA/RNA, and the protein prefers ss-DNA substrates [11]. The crystal structure of ALKBH3 [98] shows a flexible hairpin involved in flip nucleotide binding and discrimination of ss/ds-DNA [93]. ALKBH3 can repair other minor substrates such as m3T and m5C in ss/ds-DNA [22,95,96]. ALKBH3 also repairs εC [56], εA [26], 3-ethylthymine [97] and 1-ethyladenine [31].

Kinetic studies of ALKBH3 are often performed in parallel with ALKBH2. For example, ALKBH3 repairs ss-DNA m1A (k_cat_/K_m_ = 982.4 min^−1^ μM^−1^) and m3C (k_cat_/K_m_ = 763.0 min^−1^ μM^−1^) [59]. For using 2-OG as co-substrate, ALKBH3 repairs ss-m1A (k_cat_/K_m_ = 0.51 min^−1^ μM^−1^) and ss-m3C (k_cat_/K_m_ = 0.87 min^−1^ μM^−1^); while using DNA as substrate, ALKBH3 repairs ss-m1A (k_cat_/K_m_ = 0.52 min^−1^ μM^−1^) and ss-m3C (k_cat_/K_m_ = 1.03 min^−1^ μM^−1^) [61].

Researchers revealed that ALKBH3 repairs m1A in ss-RNA (k_cat_/K_m_ = 3.13 min^−1^ μM^−1^) [62]. ALKBH3 also repairs ss-m1A in RNA (k_cat_/K_m_ = 3.18 min^−1^ μM^−1^), m1A in ds-RNA (k_cat_/K_m_ = 0.39 min^−1^ μM^−1^), and m1A-probe in RNA (k_cat_/K_m_ = 2.1 min^−1^ μM^−1^) [60]. ALKBH3 can promote cancer progression through demethylating m1A in tRNA, which is more easily cleaved by protein angiogenin into tRNA-derived small RNAs when demethylated. The following binding of the fragments to Cytochrome *c* prevents apoptosis [99]. ALKBH3 catalyzes demethylation of m1A and m3C in tRNA. Michaelis–Menten steady-state kinetic studies of ALKBH3 have been performed in the stem-loop structure of RNA probes that mimic TΨC loops of tRNA. The results show that ALKBH3 quickly demethylates m1A and m3C of tRNA in vitro, which is similar to the m1A demethylation activity of ALKBH1 [91]. ALKBH3 can bind with ASCC3, which is the biggest subunit of ASCC (activating signal cointergrator complex), which can counter alkylation damage [100]. And ALKBH-mediated DNA dealkylation repair has shown improved kinetics after ASCC3 binding [101].

### 2.4. ALKBH5

ALKBH5 proteins are partially localized in nuclear speckles and have been shown to function as an m6A RNA demethylase besides FTO [64]. m6A modifications are notably distributed within the RR(m6A)CU consensus motif, where R represents G or A [102,103]. ALKBH5 also has been reported to repair *N*^6^,*N*^6^-dimethyladenine [104]. Kinetic studies show that ALKBH5 demethylates m6A (k_cat_/K_m_ = 0.12 min^−1^ μM^−1^) [64]. ALKBH5 has been correlated to FTO since they operate on the same substrate m6A in RNA. FTO and ALKBH5 have been reported to be strongly transcript-specific. Demethylation for different sequences can range from 1% to 46% for ALKBH5 catalyzed demethylation of m6A, where the sequence-containing consensus motif demonstrated high activity. Note that duplex-hairpin structures of the substrate can significantly decrease activity. Four sequences were selected to perform further steady-state kinetic studies (k_cat_/K_m_ = 0.053 to 0.098 min^−1^ μM^−1^) [67]. It has been reported that ALKBH5 demethylates m6A in ss-RNA, yielding Km = 0.192 μM, and k_cat_/K_m_ = 0.12 min^−1^ μM^−1^ was obtained with 2-OG as co-substrate [66]. One kinetic study investigated m6A repair by ALKBH5 in ss-RNA, while also considering the effect of NADP and its various forms on facilitating demethylation. However, there is no evidence that NADP can enhance ALKBH5 activity. The kinetic value of ALKBH5 repair of m6A is k_cat_/K_m_ = 0.23 min^−1^ μM^−1^ [68]. Another kinetic study of ALKBH5 repair of m6A obtained k_cat_/K_m_ = 0.11 min^−1^ μM^−1^ [65]. In the same kinetic experiment mentioned in the ALKBH3 part, they also tested ALKBH5 kinetic parameters, and the results show that ALKBH5 repairs m6A (k_cat_/K_m_ = 0.098 min^−1^ μM^−1^) [62]. Furthermore, the activity of ALKBH5 on m6A was measured when researchers pursued different fusion tags to increase heterologous expression and solubility of ALKBH5 within *E. coli* [69]. A novel fusion tag EIN (the N-terminal domain of bacterial enzyme I) was applied for recombinant expression of the human RNA demethylases ALKBH5 and FTO. The tag dramatically increased the solubility of the protein and was easily removed by proteases. A kinetic study was performed to evidence that the enzymes were active. These ALKBH5 proteins demethylate m6A (k_cat_/K_m_ = 1.6 min^−1^ μM^−1^) [69]. Most recently, two selective, novel inhibitors were found for ALKBH5, and the authors also tested the kinetics of m6A demethylation by using 2-OG as co-substrate (k_cat_/K_m_ = 18.57 min^−1^ μM^−1^) [105].

### 2.5. FTO

FTO is a protein that is associated with human obesity through a gene-finding strategy [106]. It was later determined that FTO is an 2-OG dependent dioxygenase that can repair m3T and 3-methyluracil [72,107]. FTO was also reported to demethylate m6A, which is partially localized in nuclear speckles [33]. Later, FTO has been reported to repair m6A_m_ better than m6A kinetically [70]. Most recently, FTO was found to repair m1A and m3C in tRNA [108].

The oxidation of m6A by FTO generates *N*^6^-hydroxymethyladenosine (hm6A) and *N*^6^-formyladenosine (f6A) intermediates [109]. Although FTO and ALKBH5 share similar conserved active sites [110], it has been reported that ALKBH5 does not generate these intermediates. Computational studies demonstrated how conformational dynamics influences the substrate binding [111] and catalytic mechanism of FTO [112].

#### 3-Methyluracil and 3-methythymine.

FTO was shown to catalyze the demethylation of m3U in ss-RNA (k_cat_/K_m_ = 0.014 min^−1^ μM^−1^) with higher efficiency than m3T in ss-DNA (k_cat_/K_m_ = 0.007 min^−1^ μM^−1^) [72]. 2-OG was also identified as a co-substrate for FTO repair of m3U in an effort to determine whether FTO is a sensor for 2-OG levels. The authors used a stem-loop substrate containing m3U with FAM (6-carboxyfluorescein), which can be cleaved by RNAse after the demethylation of m3U to uracil. The K_m_ of 2-OG was found to be 2.88 μM, which is 10-fold lower than the estimated intracellular condition. The result shows that FTO is unlikely to be a sensor for 2-OG [71].

#### *N*^6^,2′-O-dimethyladenosine.

The kinetics of FTO demethylating m6A_m_ were studied for m6A_m_ adjacent to the m7G cap. mRNA can be methylated at the 2′-hydroxyl position of the ribose sugar [113]. If the nucleotide followed by m7G with a triphosphate link at 5′ end of mRNAs is 2′-O-methyladenosine (A_m_), it can be methylated further into m6A_m_ [114]. FTO can demethylate m6A_m_ with k_cat_/K_m_ = 6.55 min^−1^ μM^−1^ [70].

#### *N*^6^-methyladenine.

FTO as an RNA demethylase has efficient oxidative demethylation activity targeting the m6A residues in RNA in vitro (k_cat_/K_m_ = 0.724 min^−1^ μM^−1^) [33]. NADP has been shown to strongly bind to FTO and enhance FTO-mediated m6A demethylation in vitro and in vivo. Different forms of NADP derivatives and cofactor vitamin C have been added to the reaction mixture, exhibiting different capabilities to increase FTO activity, implying that FTO is potentially involved in the regulation of the cellular redox state [68]. The authors also found that NADP exerts much less effect on ALKBH5 than FTO, implying distinct regulatory mechanisms for ALKBH5 and FTO. For FTO demethylation of m6A in ss-RNA, the kinetic parameters were reported in the presence of 50 μM cofactors, NADPH (k_cat_/K_m_ = 1.01 min^−1^ μM^−1^), NADH (k_cat_/K_m_ = 0.55 min^−1^ μM^−1^), NADP^+^ (k_cat_/K_m_ = 0.29 min^−1^ μM^−1^), NAD^+^ (k_cat_/K_m_ = 0.20 min^−1^ μM^−1^), and vitamin C (k_cat_/K_m_ = 0.045 min^−1^ μM^−1^) [68]. For comparison, FTO repairs m6A under regular conditions (2 mM vitamin C) with k_cat_/K_m_ = 0.78 min^−1^ μM^−1^ [68]. As mentioned above in the ALKBH5 section, m6A modifications are typically allocated within the RR(m6A)CU consensus motif. Sequence-dependent kinetic measurements were also performed for FTO demethylation of m6A; here, demethylation activities for various sequences range from 2% to 78%. Four sequences were selected to run further steady-state kinetic studies, and their kinetic data range is 0.39 to 0.77 min^−1^ μM^−1^ for k_cat_/K_m_ [67]. In the previously mentioned tags fusion study, FTO demethylation of m6A was also measured for its kinetic parameters: untagged FTO demethylates m6A with k_cat_/K_m_ = 0.69 min^−1^ μM^−1^ [62]. Other FTO oxidizing m6A abilities were also tested: k_cat_/K_m_ = 0.0013 min^−1^ μM^−1^ [69] and k_cat_/K_m_ = 0.63 min^−1^ μM^−1^ [65]. Researchers also found that FTO demethylates regular m6A at the GGm6ACU consensus motif (k_cat_/K_m_ = 0.06 min^−1^ μM^−1^) and m6A next to the m7G triphosphate cap (k_cat_/K_m_ = 0.07 min^−1^ μM^−1^) [70].

### 2.6. Ten-Eleven Translocation and J-Binding Protein (TET/JBP) Proteins

The first characterized TET/JBP family protein is JBP. It can catalyze the hydroxylation of thymine in DNA into hm5U as the first step of synthesizing base J [115,116]. It is found in the kinetoplastid flagellates, such as pathogenic *Trypanosoma* and *Leishmania* species [117,118], but is absent from eukaryotes, prokaryotes, and viruses [43].

TET proteins (TET1, TET2, and TET3) are identified as mammalian homologs of the trypanosome protein JBP [35]. Experiments show that TET proteins are 2-OG dependent enzymes that successively oxidize 5-methylcytosine into 5-hydroxymethylcytosine, 5-formylcytosine and 5-carboxylcytosine [34]. TET2 has been studied for its kinetic activity. The results show that TET2 is more active on m5C (k_cat_/K_m_ = 0.27 min^−1^ μM^−1^) than hm5C (k_cat_/K_m_ = 0.042 min^−1^ μM^−1^) and f5C (k_cat_/K_m_ = 0.021 min^−1^ μM^−1^). hm5C is less prone to be further oxidized than m5C, suggesting that hm5C could be a potential stable biomarker for regulatory functions [63]. Until now, there has been no report on the kinetic properties of TET1 and 3. Computational studies elaborated on the catalytic mechanism of TET2 and the effects of clinically important mutations on the reaction mechanism as well as its reaction with unnatural alkylated substrates [119–122].

### 2.7. AlkB

AlkB is one of four enzymes in *E. coli* that respond to alkylation damage during the adaptive response [123]. The AlkB structure [74] shows that this enzyme uses a base-flipping mechanism to detect the damaged base [92]. Various substrates of AlkB have been reported and studied [44,124]. Computational studies revealed the catalytic mechanism of AlkB with a variety of alkylated substrates [20,125–128]. Importantly, studies reveal the effects of the nature of the substrate (ss- vs. ds-DNA) and the influence of long-range interactions and enzyme and substrate dynamics on the reaction mechanism [20].

#### 1-Methyladenine.

m1A and m3C were found to be the major substrates of AlkB. The kinetic study of AlkB repair of m1A was first performed using various substrates: minimal and extended substrate, poly(dA) containing m1A, short DNA oligonucleotides, and nucleotide triphosphate [73]. The authors found that long DNA substrates have better activity than short oligonucleotides. The minimal substrate for AlkB is 1-me-dAMP(5′) (k_cat_/K_m_ = 0.80 min^−1^ μM^−1^), while the trimer dTm1AT has a high kinetic value (k_cat_/K_m_ = 2.6 min^−1^ μM^−1^). In addition, substrates lacking a phosphate at the 5′ position to the lesion are poor substrates for demethylation: compare 1-me-dAMP (3′) (k_cat_/K_m_ = 0.2 min^−1^ μM^−1^) to 1-me-dAMP (5′) (k_cat_/K_m_ = 2.1 min^−1^ μM^−1^) [73]. Later, another study tested m1A demethylation with the same trimer sequence, dTm1AT, and obtained similar results (k_cat_/K_m_ = 3.7 min^−1^ μM^−1^) [74]. Another study used formaldehyde dehydrogenase to convert the byproduct formaldehyde to formic acid and monitor the generation of an NADH analog using fluorescence. The results show that AlkB demethylates ds-1mA (k_cat_/K_m_ = 0.48 min^−1^ μM^−1^) and ss-m1A (k_cat_/K_m_ = 0.68 min^−1^ μM^−1^) with comparable efficiencies, and that the enzyme only has a modest preference for ss-DNA substrates [75]. In these experiments, differences in nucleic acid substrate length, the binding of diverse substrates, and coupling between successive chemical steps in the reaction cycle could account for the varying results, indicating that accessory factors could potentially influence the recognition of damaged bases in vivo. AlkB protein repairs the trimer Tm1AT (k_cat_/K_m_ = 1.9 min^−1^ μM^−1^) much less efficiently compared to the pentamer CAm1AAT (k_cat_/K_m_ = 97.0 min^−1^ μM^−1^) [76]. A kinetic study was performed investigating the dynamic conformational transitions of AlkB. The authors found that the key conformational transition controlling the catalytic cycle of AlkB involves movement of the nucleotide recognition lid that interacts with the Fe(II)/2-OG core. Their results show that AlkB repairs m1A with k_cat_/K_m_ = 1.6 min^−1^ μM^−1^ [77]. Moreover, active site residues were identified and mutated and the results show different demethylation activities: k_cat_/K_m_ = 3.7 min^−1^ μM^−1^ for unmodified AlkB repairing m1A in DNA, k_cat_/K_m_ = 1.6 min^−1^ μM^−1^ for repair in RNA, k_cat_/K_m_ = 0.018 min^−1^ μM^−1^ for AlkB D135N, k_cat_/K_m_ = 0.030 min^−1^ μM^−1^ for AlkB E136L, and k_cat_/K_m_ = 0.035 min^−1^ μM^−1^ for AlkB D135L [65]. In the previously mentioned self-complementary probe using the scanning fluorimetry technique to study ALKBH2, the authors also tested m1A repair by AlkB and found k_cat_/K_m_ = 0.63 min^−1^ μM^−1^; ss-m1A repair in RNA gave k_cat_/K_m_ = 2.6 min^−1^ μM^−1^; and m1A repair in ds-RNA gave k_cat_/K_m_ = 3.2 min^−1^ μM^−1^ [60]. For AlkB repair of ss-m1A, k_cat_/K_m_ = 0.59 min^−1^ μM^−1^ was found; and for m1A in ds-DNA, k_cat_/K_m_ = 0.38 min^−1^ μM^−1^ was determined [61]. Recently, Baldwin et al. used a transient-state kinetic analysis for single turnover reactions to determine the elementary steps of the enzymatic mechanism. Their kinetic data show a faster rate than previously reported. In short, this method requires rapid mixing of a substrate with sufficient enzyme to directly observe intermediates and products formed in one single reaction cycle. The advantage of this method is that it avoids the problem of self-inactivation of dioxygenases during multiple turnovers. The transient-state kinetics analysis also shows that AlkB can repair m1A preferentially in single-stranded DNA (k_cat_/K_m_ = 1317.1 min^−1^ μM^−1^) over double-stranded DNA (k_cat_/K_m_ = 71.1 min^−1^ μM^−1^) [78].

#### 3-Methylcytosine.

m3C was also tested by the method of formaldehyde dehydrogenase converting formaldehyde to formic acid and monitoring the generation of an NADH analog using fluorescence. The results show that AlkB demethylates m3C in ds-DNA (k_cat_/K_m_ = 0.35 min^−1^ μM^−1^) and ss-m3C (k_cat_/K_m_ = 0.65 min^−1^ μM^−1^) [75]. Other studies used an approach to directly quantitate DNA substrates and products that differ by a single methyl group, based on capillary electrophoresis with laser-induced fluorescence detection. This study achieved baseline separation of a 15mer nucleotide with a fluorescence label and a single m3C unit and obtained k_cat_/K_m_ = 73.6 min^−1^ μM^−1^ [80]. In the study on the dynamic conformational transitions of AlkB, similar kinetic results were obtained for AlkB repair of m3C (k_cat_/K_m_ = 53.0 min^−1^ μM^−1^) [77]. AlkB kinetic parameters for repairing m3C in ss-DNA (k_cat_/K_m_ = 1.2 min^−1^ μM^−1^) and ds-DNA (k_cat_/K_m_ = 0.76 min^−1^ μM^−1^) were also reported [61]. Nigam et al. found that AlkB inefficiently repairs m3C in long ss-DNA but readily repairs single-stranded DNA binding protein (SSB)-bound methylated ss-DNA of equal length. The 70mer poly T was used as the DNA sequence, with m3C present in different positions in the sequence, and their AlkB efficiency was tested with SSB. m3C at position 15 shows the highest activity (K_m_ = 8.2 μM^−1^) over the m3C at position 1 (K_m_ = 2.4 μM^−1^), a location that could potentially be wrapped by SSB [81].

#### 1,*N*^6^-ethenoadenine.

εA is one of the etheno-DNA lesions, which are exocyclic DNA lesions usually formed by exposure to either lipid peroxidation (LPO) products or the carcinogen vinyl chloride [56]. In *E. coli*, it is repaired by direct reversal repair (AlkB) and base excision repair (AAG, alkyladenine DNA glycosylase). The repair of εA by AlkB was initially reported in 2005 [25,26].

Kinetic studies of AlkB repairing εA have also been carried out by different laboratories with various methods. Yu et al. performed a kinetic study of AlkB repairing the TεAT trimer and found k_cat_/K_m_ = 0.002 min^−1^ μM^−1^ [76]. Gururaj et al. detected glyoxal, a byproduct of εA repair, by reaction with 2-hydrazinobenzothiazole, forming a yellow-colored compound with a distinct absorption spectrum with an absorption band at 365 nm. Their results show that a 40mer containing adenine, treated by chloroacetaldehyde to form εA, is repaired by AlkB with k_cat_/K_m_ = 0.0019 min^−1^ μM^−1^ [82]. As mentioned in the above section of m1A, a transient state kinetic study shows that AlkB can repair εA in ss-DNA (k_cat_/K_m_ = 8.5 min^−1^ μM^−1^) and ds-DNA (k_cat_/K_m_ = 12.1 min^−1^ μM^−1^) [78].

#### *N*^6^-methyladenine.

AlkB has a relatively low activity to m6A compared to m1A and m3C. Zhu et al. performed kinetic studies of m6A repair by wild-type and mutant AlkB. The unmodified AlkB shows k_cat_/K_m_ = 0.007 min^−1^ μM^−1^, whereas the AlkB variants having swapped sequences from FTO and ALKBH5 show improved activity towards m6A [65].

#### 1-Methylguanine.

AlkB has been reported to repair m1G with weak activity. The Pan group identified the AlkB D135S variant, which has higher reactivity than the wild-type enzyme for repairing m1G. Aspartic acid 135 has been reported to be able to form H-bonds with the N6 group of adenine and N4 group of cytosine [74]. A high throughput platform was used to evaluate variants of AlkB. AlkB variant D135T has the highest activity toward m1G (k_cat_/K_m_ = 15.7 ± 3.7 min^−1^ μM^−1^) [79]. The results reveal that these positively charged substrates are favorably positioned in the active site by interacting with the negatively charged carboxylate group of D135; the shorter side chain of D135S seems to allow more room to accommodate m1G while sustaining the crucial hydrogen bond with m1G, which may explain the improvement in activity. The engineered variant of AlkB D135V/L118V can covert *N*^2^,*N*^2^-dimethylguanosine (m22G) into m2G [129].

### 2.8. Other Mammalian Homologs of AlkB

ALKBH4, ALKBH7, and ALKBH8 have been tested with respect to their demethylation activities. ALKBH4 demethylates m6A in DNA; it can also mediate the demethylation of a monomethylated site in actin (K84me1), which controls the actin–myosin interaction and other actomyosin-dependent processes, such as cytokinesis and cell migration [130]. ALKBH6 in humans is localized in the nucleus and cytoplasm; the substrate of ALKBH6 has yet to be discovered [131,132]. ALKBH7 is nuclear-encoded but is primarily localized in the mitochondria of mammalian cells. ALKBH7 demethylates m22G and m1A within mitochondrial Ile and Leu1 pre-tRNA regions [133]. ALKBH7 has also been reported to repair εA and m3C [134]. ALKBH8 is exclusively located in the cytoplasm and has three domains: the N-terminal RNA recognition motif, the middle 2-OG/Fe(II)-dependent AlkB-like domain, and the C-terminal methyltransferase domain. Its structure and hydroxylation of 5-methoxycarbonylmethyluridine (mcm5U) suggest a potential role in the regulation of posttranscriptional tRNA modification through methylation/demethylation [135].

## 3. Conclusions

In this review, we inspect the AlkB and TET family proteins, mainly focusing on their kinetic behaviors. Many researchers have shown that these enzymes oxidize various substrates in DNA and RNA. Kinetic studies have provided important information with respect to the mechanism of an enzyme-catalyzed reaction by determining the rate of reaction and how the rate changes in response to changes in experimental parameters. Among the kinetic parameters, the ratio of k_cat_/K_m_ provides a reliable measure of catalytic efficiency.

A useful application of kinetic studies is to compare repair efficiency of different DNA/RNA substrates (modified bases). Some examples are listed below. Studies show that ALKBH2 and ALKBH3 prefer repairing m1A/m3C over m3T, and ALKBH3 prefers to repair lesions in ss-DNA/RNA, while ALKBH2 prefers to repair lesions in ds-DNA [95]. There is more information from the kinetic studies that can further help us understand the reaction mechanism. For instance, FTO was first discovered to repair m3T and m3U, but the kinetic parameters are relatively low compared to other ALKBH family proteins [72,107]; later, m6A was discovered as a better substrate [33]. Kinetics is also a tool to find out about the property of a protein in vivo. In the case of NADPH modulating FTO, the results imply that FTO is involved in the regulation of the cellular redox state, while there is no such effect for ALKBH5 [68]. 2-OG was also measured as a substrate for FTO to repair the m3U lesion; the kinetic results show FTO is a sensor for cellular 2-OG levels [71]. TET2 is more efficient in oxidizing m5C than hm5C, suggesting that TET2 is possibly evolutionarily tuned and hm5C is a potential stable marker with regulatory functions [63]. Kinetics also show the sequence-dependent activities of enzymes, such that ALKBH5 and FTO prefer the GG(m6A)CU consensus sequence. In addition, substrates in duplex-hairpin structures significantly decrease enzyme activity [67].

In most kinetic studies, the conversion of an enzymatic reaction is usually lower than 20% to obtain accurate kinetic parameters. For the detection of starting material and product, many studies use LC-MS, because a repaired base usually has less MW than the starting material. HPLC with a UV detector has been widely used for detection as well, because DNA bases have strong absorbances around 260 nm in the UV spectrum, and the starting material and the product can be separated by different types of columns [136]. Sometimes it is hard to detect the amount of product DNA; in such cases, researchers have used other co-products, for example formaldehyde and glyoxal (Figure 1), to monitor the reactions. A research group used formaldehyde dehydrogenase to convert formaldehyde to formic acid and monitored the formation of an NADH analog using fluorescence [75]. Gururaj et al. used glyoxal, a byproduct of εA repair, and reacted it with 2-hydrazinobenzothiazole, which forms a complex with a yellow color that has a distinct absorption spectrum with a band at 365 nm [82].

For an individual enzyme, there could be several DNA/RNA substrates. How to differentiate the repair efficiency among them needs a kinetic study carried out under the same condition for all substrates. In the literature, different methods provide different kinetic parameters even for the same enzyme and substrate. The data from different sources may not be able to distinguish the differences between these substrates. For example, it was initially reported that ALKBH2 repairs m1A in ds-DNA with k_cat_/K_m_ = 2572.5 min^−1^ μM^−1^ and m3C with k_cat_/K_m_ =3176 min^−1^ μM^−1^, compared to m1A in single-stranded DNA (k_cat_/K_m_ = 1100 min^−1^ μM^−1^) and m3C in ss-DNA (k_cat_/K_m_ = 775 min^−1^ μM^−1^) [59]. These numbers are quite different from later published results: ALKBH2 repairs m1A in ds-DNA (k_cat_/K_m_ = 0.74 min^−1^ μM^−1^) and ss-m1A (k_cat_/K_m_ = 0.25 min^−1^ μM^−1^ [61]. These differences are possibly due to the fact that the earlier results were obtained by using restriction enzymes to cut the repaired oligomers for measuring the demethylation. Additionally, the reaction time course was monitored at a single time point, 1 h, which is typically long for kinetic studies. Single turnover reactions could generate kinetic data that are faster than data from steady-state kinetics [78]. These examples demonstrate the necessity of performing the kinetic studies of an enzyme with all of its substrates under the same condition. The 2-OG/Fe(II)-dependent enzymes discussed in this review are important in genetics and epigenetics [61]. However, many substrates of certain enzymes are still not clear, and additionally, in many cases kinetic data are not available. One of the future goals for this research field is to study the mechanism and kinetic behavior of these proteins in a more detailed manner, and then apply this information to determine their functions in biology and disease.

## Figures and Tables

**Table 1.** Updated DNA/RNA substrates of 2-OG/Fe(II)-dependent enzymes.

Enzyme	Substrate
AlkB	**DNA**: m1A, m3C, m1G, m3T, m4C, m2G, m22G, m5C, e1A, εA, εC, 1,*N*^2^-εG, e2G, EA, FF, HF, *α*HOPG, *γ*HOPG, M1G, HEC, HPC
	**RNA**: m1A, m3C, m1G
ALKBH1	**DNA**: m3C, m6A
	**RNA**: m3C, m5C, m1A
ALKBH2	**DNA**: m1A, m3C, m1G, m3T, m5C, e1A, e3T, εA, εC, 1,*N*^2^-εG
ALKBH3	**DNA**: m1A, m3C, m3T, m5C, e1A, e3T, εC, εA
	**RNA**: m1A, m3C, m6A
ALKBH4	**DNA**: m6A
ALKBH5	**RNA**: m6A, m66A
ALKBH6	–
ALKBH7	**RNA**: m1A, m3C, m22G, εA
ALKBH8	**RNA**: mc5mU
FTO	**DNA**: m3T, m6A
	**RNA**: m3U, m6A, m1A, m3C
TET1–3	**DNA**: m5C, T
	**RNA**: m5C

**Table 2.** Kinetic parameters of ALKBH1, 2, 3 and TET2 for different substrates (X: modified base).

Enzyme	DNA/RNA	Substrate	DNA/RNA Sequence 5′−3′	K_cat_ (min^−1^)	Km (μm)	K_cat_/K_m_ (min^−1^ μm^−1^)	Reference
**ALKBH1**	DNA	m6A	ACCTTATGGAXAGCATGCTTG in ds-DNA	0.136 ± 0.0036	3.18 ± 0.28	0.04	[58]
	DNA		ACCTTATGGAXAGCATGCTTG	0.076 ± 0.0012	2.79 ± 0.18	0.03	
**ALKBH2**	DNA	m1A	AAAGCAGXATTCGAAAAAGCGAAA in ds-DNA	823.2 ± 120	0.320 ± 0.073	2573	[59]
	DNA		AAAGCAGXATTCGAAAAAGCGAAA	198 ± 16.2	0.183 ± 0.023	1082	
	RNA		AAAGCAGXAUUCGAA in ds-RNA	2.19 ± 0.05	0.30 ± 0.07	7.4	[60]
	RNA		CGCGXAUUCGCG	3.67 ± 0.35	1.09 ± 0.14	3.4	
	RNA		AAAGCAGXAUUCGAA	4.07 ± 0.15	0.95 ± 0.11	4.3	
	DNA		GAAGACCTXGGCGTCC in ds-DNA	2.5 ± 0.1	7.3 ± 0.9	0.34	[61]
	DNA		GAAGACCTXGGCGTCC	1.1 ± 0.1	4.1 ± 0.9	0.27	
	DNA	m3C	AAAGCACXGGTCGAAAAAGCGAAA in ds-DNA	530.4±52.8	0.167 ± 0.027	3176	[59]
	DNA		AAAGCACXGGTCGAAAAAGCGAAA	63.6±7.2	0.0822 ± 0.022	774	
	DNA		GAAGACCTXGGCGTCC in ds-DNA	2.6 ± 0.1	1.9 ± 0.4	1.3	[61]
	DNA		GAAGACCTXGGCGTCC	1.7 ± 0.1	1.4 ± 0.2	1.2	
**ALKBH3**	DNA	m1A	AAAGCAGXATTCGAAAAAGCGAAA in ds-DNA	109.8±2.28	0.263 ± 0.110	418	[59]
	DNA		AAAGCAGXATTCGAAAAAGCGAAA	178.8±44.4	0.182 ± 0.140	982	
	RNA		AAAGCAGXAUUCGAA in ds-RNA	2.57 ± 0.27	6.60 ± 0.19	0.39	[60]
	RNA		AAAGCAGXAUUCGAA	3.56 ± 0.31	1.12 ± 0.16	3.2	
	RNA		CGCGXAUUCGCG	3.13 ± 0.22	1.47 ± 0.08	2.1	
	DNA		GAAGACCTXGGCGTCC	1.2 ± 0.0	2.3 ± 0.1	0.51	[61]
	DNA		AAAGCAGXATTCGAA	3.04 ± 0.22	0.97 ± 0.07	3.1	[62]
	DNA	m3C	AAAGCACXGGTCGAAAAAGCGAAA in ds-DNA	2.268±0.462	0.0084 ± 0.016	270	[59]
	DNA		AAAGCACXGGTCGAAAAAGCGAAA	123.6±19.2	0.162 ± 0.048	763	
	DNA		GAAGACCTXGGCGTCC	1.7 ± 0.0	1.9 ± 0.4	0.87	[61]
**TET2**	DNA	m5C	ACCACXGGTGGT	0.127 ± 0.019	0.48 ± 0.19	0.27	[63]
	DNA	hm5C	ACCACXGGTGGT	0.038 ± 0.005	0.90 ± 0.30	0.04	
	DNA	f5C	ACCACXGGTGGT	0.0276 ± 0.002	1.30 ± 0.27	0.02	

**Table 3.** Kinetic parameters of ALKBH5 and FTO for different substrates (X: modified base).

Enzyme	DNA/RNA	Substrate	DNA/RNA Sequence 5′−3′	K_cat_ (min ^1^)	Km (μm)	K_cat_/K_m_ (min^−1^ μm^−1^)	Reference
**ALKBH5**	RNA	m6A	AUUGUCAXCAGCAGC	0.169 ± 0.0106	1.38 ± 0.2653	0.12	[64]
	DNA		ATTGTCAXCAGCAGA	0.174 ± 0.008	1.66 ± 0.16	0.11	[65]
	RNA		UACACUCGAUCUGGXCUAAAGCU GCUC-biotin-3′	0.3 ± 0.067	2.5 ± 0.5	0.12	[66]
	RNA		UACACUCGAUCUGGXCUAAAGCU GCUC-biotin-3′		0.192		
	RNA		GGXCU	0.140 ± 0.013	2.344 ± 0.140	0.06	[67]
	DNA		GAXCA	0.162 ± 0.014	2.251 ± 0.042	0.07	
	RNA		GCGGXCUCCAGAUG	0.172 ± 0.010	1.755 ± 0.088	0.1	
	RNA		CCCCXCCCCCCCCC	0.137 ± 0.021	2.583 ± 0.256	0.05	
	RNA		GGXCU	0.16 ± 0.02	1.64 ± 0.05	0.1	[62]
	RNA		AUUGUCAXCAGCAG	0.306 ± 0.034	1.335 ± 0.213	0.23	[68]
	DNA		GGXCT	2.6 ± 0.6	1.6 ± 0.1	1.6	[69]
**FTO**	RNA	m6A	AUUGUCAXCAGCAGC	0.296 ± 0.004	0.409 ± 0.023	0.72	[33]
	RNA		AUUGUCAXCAGCAGC	0.381 ± 0.114	0.6 ± 0.12	0.63	[65]
	RNA		m7GpppXCA	7.77	16.09	0.48	[70]
	RNA		m7GpppACX	0.46	6.4	0.07	
	RNA		GGXCU	0.54	9.29	0.06	
	RNA		GGXCU	0.347 ± 0.015	0.508 ± 0.126	0.68	[67]
	DNA		GGXCT	0.334 ± 0.57	0.586 ± 0.137	0.57	
	DNA		GCGGXCUCCAGAUG	0.376 ± 0.009	0.488 ± 0.074	0.77	
	RNA		CCCCXCCCCCCCCC	0.268 ± 0.012	0.688 ± 0.025	0.39	
	RNA		GGXCU	0.35 ± 0.03	0.51 ± 0.06	0.69	[62]
	RNA		AUUGUCAXCAGCAG	0.46 ± 0.055	0.59 ± 0.094	0.78	[68]
	RNA		containing 50 μM NADPH	0.406 ± 0.0467	0.401 ± 0.0521	1.01	
			50 μM NADH	0.290 ± 0.0311	0.528 ± 0.0660	0.55	
			50 μM NADP+	0.282 ± 0.0340	0.961 ± 0.127	0.29	
			50 μM NAD+	0.224 ± 0.0291	1.125 ± 0.158	0.20	
			50 μM Vc	0.136 ± 0.0258	3.015 ± 0.572	0.045	
	DNA		GGXCT	0.015 ± 0.005	12 ± 2	0.001	[69]
	RNA	m6A_m_	m7GpppX	8.78	1.34	6.55	[70]
	RNA	m3U	CTGACGGAGAXGAACGTCAG		2.88		[71]
	RNA		CUUGUCAXCAGCAGA	0.115 ± 0.022	8.51 ± 3.13	0.014 ± 0.007	[72]
	DNA	m3T	CTTGTCAXCAGCAGA	0.007 ± 0.0002	0.95 ± 0.12	0.007 ± 0.002	[72]

**Table 4.** Kinetic parameters of *E. coli* AlkB for different substrates (X: modified base).

Enzyme	DNA/RNA	Substrate	DNA/RNA Sequence 5′−3′	K_cat_ (min ^1^)	Km (μM)	K_cat_/K_m_ (min^−1^ μm^−1^)	Reference
**AlkB**	DNA	m1A	poly(dA) methylated with [^14^C]MeI	11.7 ± 0.2	1.4 ± 0.2	8.6	[73]
	DNA		TXT	7.4 ± 0.6	2.8 ± 0.9	2.6	
	DNA		TX	3.7	4.4	0.8	
	DNA		TXT	2.7 ± 0.8	1.4 ± 0.5	1.9	[74]
	DNA		CGTCGXATTCTAGAGCCCC	3.7 ± 0.2	5.4 ± 0.9	0.68	[75]
	DNA		CGTCGXATTCTAGAGCCCC in ds-DNA	3.1 ± 0.2	6.2 ± 1.3	0.48	
	DNA		TXT	2.7 ± 0.8	1.4 ± 0.9	1.9	[76]
	DNA		CAXAT	5.4 ± 1.3	0.06 ± 0.01	97	
	DNA		TXT	5.2 ± 0.2	3.2 ± 0.4	1.6	[77]
	DNA		ATTGTCAXCAGCAGA	7.41 ± 0.47	2.00 ± 0.35	3.7	[65]
	RNA		AUUGUCAXCAGCAGC	3.72 ± 0.19	2.32 ± 0.31	1.6	
	RNA		AAAGCAGXAUUCGAA in ds-RNA	2.25 ± 0.18	3.56 ± 0.24	0.63	[60]
	RNA		r(CGCGXAUUCGCG) probe	4.10 ± 0.29	1.30 ± 0.12	3.2	
	RNA		AAAGCAGXAUUCGAA	3.75 ± 0.12	1.44 ± 0.25	2.6	
	DNA		GAAGACCTXGGCGTCC	4.2 ± 0.2	7.1 ± 1.1	0.59	[61]
	DNA		GAAGACCTXGGCGTCC in ds-DNA	4.8 ± 0.2	12.7 ± 1.3	0.38	
	DNA		CGATAGCATCCTXCCTTCTCTCCAT	54 ± 1.8	0.041 ± 0.007	1317	[78]
	DNA		CGATAGCATCCTXCCTTCTC in ds-DNA	46.2 ± 1.2	0.65 ± 0.05	71.1	
	DNA	m6A	ATTGTCAXCAGCAGA	0.107 ± 0.013	14.93 ± 2.46	0.01	[65]
**D135S**	RNA	m1G	GAGCXUUAG			2.2	[79]
**D135T**	RNA		GAGCXUUAG	0.052 ± 0.008	3.3 ± 1.3	15.7 ± 3.7	
	DNA	m3C	CGTCGAATTXTAGAGCCCC	2.2 ± 0.1	3.4 ± 0.6	0.65	[75]
	DNA		CGTCGAATTXTA GAGCCCC in ds-DNA	3.3 ± 0.2	9.3 ± 2.4	0.35	
	DNA		TXT	21 ± 4	24 ± 5	0.9	[76]
	DNA		CAXAT	23 + 10	0.29 ± 0.03	78.3	
	DNA		TTXTTTTTTTTTTTT	2.6 ± 0.3	0.0353 ± 0.0066	73.6	[80]
	DNA		CAXAT	21.2 ± 1.1	0.4 ± 0.1	53	[77]
	DNA		GAAGACCTXGGCGTCC in ds-DNA	8.2 ± 0.4	10.8 ± 1.9	0.76	[61]
	DNA		GAAGACCTXGGCGTCC	24.5 ± 0.7	19.9 ± 1.3	1.2	
	DNA		70-mer Poly T with X at position 1		2.4		[81]
	DNA		70-mer Poly T with X at position 35		6.7		
	DNA		70-mer Poly T with X at position 15		8.2		
	DNA	εA	TXT	0.06			[25]
	DNA		GAAGACCTXGGCGTCC	1.8			[26]
	DNA		TXT	0.13 ± 0.05	60 ± 14	0.002	[76]
	DNA		40-mer containing A treated by chloroacetaldehyde	0.134	67.4	0.0019	[82]
	DNA		CGATAGCATCCTXCCTTCTCTCCAT	45 ± 6.6	5.3 ± 1.3	8.5	[78]
	DNA		CGATAGCATCCTXCCTTCTC in ds-DNA	102 ± 30	8.4 ± 3.4	12.1	

## References

[1] Rydberg B; Lindahl T Nonenzymatic Methylation of DNA by the Intracellular Methyl Group Donor S-Adenosyl-L-Methionine Is a Potentially Mutagenic Reaction. EMBO J. 1982, 1, 211–216. 7188181 10.1002/j.1460-2075.1982.tb01149.x PMC553022

[2] Mathison BH; Frame SR; Bogdanffy MS DNA Methylation, Cell Proliferation, and Histopathology in Rats Following Repeated Inhalation Exposure to Dimethyl Sulfate. Inhal. Toxicol 2004, 16, 581–592. 16036751 10.1080/08958370490464553

[3] Stone MP; Cho Y-J; Huang H; Kim H-Y; Kozekov ID; Kozekova A; Wang H; Minko IG; Lloyd RS; Harris TM; Interstrand DNA Cross-Links Induced by α,β-Unsaturated Aldehydes Derived from Lipid Peroxidation and Environmental Sources. Acc. Chem. Res 2008, 41, 793–804. 18500830 10.1021/ar700246x PMC2785109

[4] Marnett L Endogenous DNA Damage and Mutation. Trends Genet. 2001, 17, 214–221. 11275327 10.1016/s0168-9525(01)02239-9

[5] Bordin DL; Lirussi L; Nilsen H Cellular Response to Endogenous DNA Damage: DNA Base Modifications in Gene Expression Regulation. DNA Repair 2021, 99, 103051. 33540225 10.1016/j.dnarep.2021.103051

[6] Barrows LR; Magee PN Nonenzymatic Methylation of DNA by S-Adenosylmethionine in Vitro. Carcinogenesis 1982, 3, 349–351. 7083475 10.1093/carcin/3.3.349

[7] Sedgwick B; Lindahl T Recent Progress on the Ada Response for Inducible Repair of DNA Alkylation Damage. Oncogene 2002, 21, 8886–8894. 12483506 10.1038/sj.onc.1205998

[8] Lindahl T; Sedgwick B; Sekiguchi M; Nakabeppu Y Regulation and Expression of the Adaptive Response to Alkylating Agents. Annu. Rev. Biochem 1988, 57, 133–157. 3052269 10.1146/annurev.bi.57.070188.001025

[9] Mielecki D; Grzesiuk E Ada Response—A Strategy for Repair of Alkylated DNA in Bacteria. FEMS Microbiol. Lett 2014, 355, 1–11. 24810496 10.1111/1574-6968.12462 PMC4437013

[10] Mielecki D; Wrzesiński M; Grzesiuk E Inducible Repair of Alkylated DNA in Microorganisms. Mutat. Res./Rev. Mutat. Res 2015, 763, 294–305. 25795127 10.1016/j.mrrev.2014.12.001

[11] Aas PA; Otterlei M; Falnes PØ; Vågbø CB; Skorpen F; Akbari M; Sundheim O; Bjørås M; Slupphaug G; Seeberg E; Human and Bacterial Oxidative Demethylases Repair Alkylation Damage in Both RNA and DNA. Nature 2003, 421, 859–863. 12594517 10.1038/nature01363

[12] Aravind L; Koonin EV The DNA-Repair Protein AlkB, EGL-9, and Leprecan Define New Families of 2-Oxoglutarate- and Iron-Dependent Dioxygenases. Genome Biol 2001, 2, research0007.1. 10.1186/gb-2001-2-3-research0007 PMC30706 11276424

[13] Falnes PØ; Johansen RF; Seeberg E AlkB-Mediated Oxidative Demethylation Reverses DNA Damage in *Escherichia coli*. Nature 2002, 419, 178–182. 12226668 10.1038/nature01048

[14] Trewick SC; Henshaw TF; Hausinger RP; Lindahl T; Sedgwick B Oxidative Demethylation by *Escherichia coli* AlkB Directly Reverts DNA Base Damage. Nature 2002, 419, 174–178. 12226667 10.1038/nature00908

[15] Clifton IJ; McDonough MA; Ehrismann D; Kershaw NJ; Granatino N; Schofield CJ Structural Studies on 2-Oxoglutarate Oxygenases and Related Double-Stranded β-Helix Fold Proteins. J. Inorg. Biochem 2006, 100, 644–669. 16513174 10.1016/j.jinorgbio.2006.01.024

[16] Hausinger RP Fe(II)/α-Ketoglutarate-Dependent Hydroxylases and Related Enzymes. Crit. Rev. Biochem. Mol. Biol 2004, 39, 21–68. 15121720 10.1080/10409230490440541

[17] Welford RWD; Kirkpatrick JM; McNeill LA; Puri M; Oldham NJ; Schofield CJ Incorporation of Oxygen into the Succinate Co-Product of Iron(II) and 2-Oxoglutarate Dependent Oxygenases from Bacteria, Plants and Humans. FEBS Lett. 2005, 579, 5170–5174. 16153644 10.1016/j.febslet.2005.08.033

[18] Hutton JJ; Kaplan A; Udenfriend S Conversion of the Amino Acid Sequence Gly-Pro-Pro in Protein to Gly-Pro-Hyp by Collagen Proline Hydroxylase. Arch. Biochem. Biophys 1967, 121, 384–391. 6057106 10.1016/0003-9861(67)90091-4

[19] Islam MS; Leissing TM; Chowdhury R; Hopkinson RJ; Schofield CJ 2-Oxoglutarate-Dependent Oxygenases. Annu. Rev. Biochem 2018, 87, 585–620. 29494239 10.1146/annurev-biochem-061516-044724

[20] Waheed SO; Ramanan R; Chaturvedi SS; Lehnert N; Schofield CJ; Christov CZ; Karabencheva-Christova TG Role of Structural Dynamics in Selectivity and Mechanism of Non-Heme Fe(II) and 2-Oxoglutarate-Dependent Oxygenases Involved in DNA Repair. ACS Cent. Sci 2020, 6, 795–814. 32490196 10.1021/acscentsci.0c00312 PMC7256942

[21] Delaney JC; Essigmann JM Mutagenesis, Genotoxicity, and Repair of 1-Methyladenine, 3-Alkylcytosines, 1-Methylguanine, and 3-Methylthymine in AlkB *Escherichia coli*. Proc. Natl. Acad. Sci. USA 2004, 101, 14051–14056. 15381779 10.1073/pnas.0403489101 PMC521119

[22] Koivisto P; Robins P; Lindahl T; Sedgwick B Demethylation of 3-Methylthymine in DNA by Bacterial and Human DNA Dioxygenases. J. Biol. Chem 2004, 279, 40470–40474. 15269201 10.1074/jbc.M407960200

[23] Li D; Fedeles BI; Shrivastav N; Delaney JC; Yang X; Wong C; Drennan CL; Essigmann JM Removal of N-Alkyl Modifications from N(2)-Alkylguanine and N(4)-Alkylcytosine in DNA by the Adaptive Response Protein AlkB. Chem. Res. Toxicol 2013, 26, 1182–1187. 23773213 10.1021/tx400096m PMC3748507

[24] Li D; Delaney JC; Page CM; Yang X; Chen AS; Wong C; Drennan CL; Essigmann JM Exocyclic Carbons Adjacent to the N6 of Adenine Are Targets for Oxidation by the *Escherichia coli* Adaptive Response Protein AlkB. J. Am. Chem. Soc 2012, 134, 8896–8901. 22512456 10.1021/ja3010094 PMC3363417

[25] Delaney JC; Smeester L; Wong C; Frick LE; Taghizadeh K; Wishnok JS; Drennan CL; Samson LD; Essigmann JM AlkB Reverses Etheno DNA Lesions Caused by Lipid Oxidation in Vitro and in Vivo. Nat. Struct. Mol. Biol 2005, 12, 855–860. 16200073 10.1038/nsmb996

[26] Mishina Y; Yang C-G; He C Direct Repair of the Exocyclic DNA Adduct 1,N^6^-Ethenoadenine by the DNA Repair AlkB Proteins. J. Am. Chem. Soc 2005, 127, 14594–14595. 16231911 10.1021/ja055957m PMC2432083

[27] Maciejewska AM; Poznański J; Kaczmarska Z; Krowisz B; Nieminuszczy J; Polkowska-Nowakowska A; Grzesiuk E; Kuśmierek JT AlkB Dioxygenase Preferentially Repairs Protonated Substrates: Specificity Against Exocyclic Adducts and Molecular Mechanism of Action. J. Biol. Chem 2013, 288, 432–441. 23148216 10.1074/jbc.M112.353342 PMC3537041

[28] Maciejewska AM; Ruszel KP; Nieminuszczy J; Lewicka J; Sokołowska B; Grzesiuk E; Kuśmierek JT Chloroacetaldehyde-Induced Mutagenesis in *Escherichia coli*: The Role of AlkB Protein in Repair of 3,N4-Ethenocytosine and 3,N4-α-Hydroxyethanocytosine. Mutat. Res./Fundam. Mol. Mech. Mutagen 2010, 684, 24–34. 10.1016/j.mrfmmm.2009.11.005 19941873

[29] Kurowski MA; Bhagwat AS; Papaj G; Bujnicki JM Phylogenomic Identification of Five New Human Homologs of the DNA Repair Enzyme AlkB. BMC Genom. 2003, 4, 48. 10.1186/1471-2164-4-48 PMC317286 14667252

[30] Sanchez-Pulido L; Andrade-Navarro MA The FTO (Fat Mass and Obesity Associated) Gene Codes for a Novel Member of the Non-Heme Dioxygenase Superfamily. BMC Biochem. 2007, 8, 23. 17996046 10.1186/1471-2091-8-23 PMC2241624

[31] Duncan T; Trewick SC; Koivisto P; Bates PA; Lindahl T; Sedgwick B Reversal of DNA Alkylation Damage by Two Human Dioxygenases. Proc. Natl. Acad. Sci. USA 2002, 99, 16660–16665. 12486230 10.1073/pnas.262589799 PMC139200

[32] Loos RJF; Bouchard C FTO: The First Gene Contributing to Common Forms of Human Obesity. Obes. Rev 2008, 9, 246–250. 18373508 10.1111/j.1467-789X.2008.00481.x

[33] Jia G; Fu Y; Zhao X; Dai Q; Zheng G; Yang Y; Yi C; Lindahl T; Pan T; Yang Y-G; N6-Methyladenosine in Nuclear RNA Is a Major Substrate of the Obesity-Associated FTO. Nat. Chem. Biol 2011, 7, 885–887. 22002720 10.1038/nchembio.687 PMC3218240

[34] He Y-F; Li B-Z; Li Z; Liu P; Wang Y; Tang Q; Ding J; Jia Y; Chen Z; Li L; Tet-Mediated Formation of 5-Carboxylcytosine and Its Excision by TDG in Mammalian DNA. Science 2011, 333, 1303–1307. 21817016 10.1126/science.1210944 PMC3462231

[35] Tahiliani M; Koh KP; Shen Y; Pastor WA; Bandukwala H; Brudno Y; Agarwal S; Iyer LM; Liu DR; Aravind L; Conversion of 5-Methylcytosine to 5-Hydroxymethylcytosine in Mammalian DNA by MLL Partner TET1. Science 2009, 324, 930–935. 19372391 10.1126/science.1170116 PMC2715015

[36] Ito S; Shen L; Dai Q; Wu SC; Collins LB; Swenberg JA; He C; Zhang Y Tet Proteins Can Convert 5-Methylcytosine to 5-Formylcytosine and 5-Carboxylcytosine. Science 2011, 333, 1300–1303. 21778364 10.1126/science.1210597 PMC3495246

[37] Crawford DJ; Liu MY; Nabel CS; Cao X-J; Garcia BA; Kohli RM Tet2 Catalyzes Stepwise 5-Methylcytosine Oxidation by an Iterative and *de Novo* Mechanism. J. Am. Chem. Soc 2016, 138, 730–733. 26734843 10.1021/jacs.5b10554 PMC4762542

[38] Tamanaha E; Guan S; Marks K; Saleh L Distributive Processing by the Iron(II)/α-Ketoglutarate-Dependent Catalytic Domains of the TET Enzymes Is Consistent with Epigenetic Roles for Oxidized 5-Methylcytosine Bases. J. Am. Chem. Soc 2016, 138, 9345–9348. 27362828 10.1021/jacs.6b03243

[39] Yu M; Hon GC; Szulwach KE; Song C-X; Jin P; Ren B; He C Tet-Assisted Bisulfite Sequencing of 5-Hydroxymethylcytosine. Nat. Protoc 2012, 7, 2159–2170. 23196972 10.1038/nprot.2012.137 PMC3641661

[40] Zhang L; Chen W; Iyer LM; Hu J; Wang G; Fu Y; Yu M; Dai Q; Aravind L; He C A TET Homologue Protein from Coprinopsis Cinerea (CcTET) That Biochemically Converts 5-Methylcytosine to 5-Hydroxymethylcytosine, 5-Formylcytosine, and 5-Carboxylcytosine. J. Am. Chem. Soc 2014, 136, 4801–4804. 24655109 10.1021/ja500979k PMC3985729

[41] Yu Z; Genest P-A; ter Riet B; Sweeney K; DiPaolo C; Kieft R; Christodoulou E; Perrakis A; Simmons JM; Hausinger RP; The Protein That Binds to DNA Base J in Trypanosomatids Has Features of a Thymidine Hydroxylase. Nucleic Acids Res. 2007, 35, 2107–2115. 17389644 10.1093/nar/gkm049 PMC1874643

[42] Cliffe LJ; Kieft R; Southern T; Birkeland SR; Marshall M; Sweeney K; Sabatini R JBP1 and JBP2 Are Two Distinct Thymidine Hydroxylases Involved in J Biosynthesis in Genomic DNA of African Trypanosomes. Nucleic Acids Res. 2009, 37, 1452–1462. 19136460 10.1093/nar/gkn1067 PMC2655668

[43] Borst P; Sabatini R Base J: Discovery, Biosynthesis, and Possible Functions. Annu. Rev. Microbiol 2008, 62, 235–251. 18729733 10.1146/annurev.micro.62.081307.162750

[44] Fedeles BI; Singh V; Delaney JC; Li D; Essigmann JM The AlkB Family of Fe(II)/α-Ketoglutarate-Dependent Dioxygenases: Repairing Nucleic Acid Alkylation Damage and Beyond. J. Biol. Chem 2015, 290, 20734–20742. 26152727 10.1074/jbc.R115.656462 PMC4543635

[45] Kuznetsov NA; Kanazhevskaya LY; Fedorova OS DNA Demethylation in the Processes of Repair and Epigenetic Regulation Performed by 2-Ketoglutarate-Dependent DNA Dioxygenases. Int. J. Mol. Sci 2021, 22, 10540. 34638881 10.3390/ijms221910540 PMC8508711

[46] Perry GS; Das M; Woon ECY Inhibition of AlkB Nucleic Acid Demethylases: Promising New Epigenetic Targets. J. Med. Chem 2021, 64, 16974–17003. 34792334 10.1021/acs.jmedchem.1c01694

[47] Alemu EA; He C; Klungland A ALKBHs-Facilitated RNA Modifications and de-Modifications. DNA Repair 2016, 44, 87–91. 27237585 10.1016/j.dnarep.2016.05.026 PMC5120542

[48] Kanazhevskaya LY; Smyshlyaev DA; Alekseeva IV; Fedorova OS Conformational Dynamics of Dioxygenase AlkB and DNA in the Course of Catalytically Active Enzyme–Substrate Complex Formation. Russ. J. Bioorg. Chem 2019, 45, 630–640.

[49] Ma L; Lu H; Tian Z; Yang M; Ma J; Shang G; Liu Y; Xie M; Wang G; Wu W; Structural Insights into the Interactions and Epigenetic Functions of Human Nucleic Acid Repair Protein ALKBH6. J. Biol. Chem 2022, 298, 101671. 35120926 10.1016/j.jbc.2022.101671 PMC8892091

[50] Kanazhevskaya LY; Alekseeva IV; Fedorova OS A Single-Turnover Kinetic Study of DNA Demethylation Catalyzed by Fe(II)/α-Ketoglutarate-Dependent Dioxygenase AlkB. Molecules 2019, 24, 4576. 31847292 10.3390/molecules24244576 PMC6943663

[51] Klose RJ; Bird AP Genomic DNA Methylation: The Mark and Its Mediators. Trends Biochem. Sci 2006, 31, 89–97. 16403636 10.1016/j.tibs.2005.12.008

[52] Bird A DNA Methylation Patterns and Epigenetic Memory. Genes Dev. 2002, 16, 6–21. 11782440 10.1101/gad.947102

[53] Kinde B; Gabel HW; Gilbert CS; Griffith EC; Greenberg ME Reading the Unique DNA Methylation Landscape of the Brain: Non-CpG Methylation, Hydroxymethylation, and MeCP2. Proc. Natl. Acad. Sci. USA 2015, 112, 6800–6806. 25739960 10.1073/pnas.1411269112 PMC4460470

[54] Dubin DT; Taylor RH The Methylation State of Poly A-Containing Messenger RNA from Cultured Hamster Cells. Nucleic Acids Res. 1975, 2, 1653–1668. 1187339 10.1093/nar/2.10.1653 PMC343535

[55] Bartsch H; Nair J New DNA-Based Biomarkers for Oxidative Stress and Cancer Chemoprevention Studies. Eur. J. Cancer 2000, 36, 1229–1234. 10882861 10.1016/s0959-8049(00)00095-2

[56] Zdżalik D; Domańska A; Prorok P; Kosicki K; van den Born E; Falnes PØ; Rizzo CJ; Guengerich FP; Tudek B ˙ Differential Repair of Etheno-DNA Adducts by Bacterial and Human AlkB Proteins. DNA Repair 2015, 30, 1–10. 25797601 10.1016/j.dnarep.2015.02.021 PMC4451939

[57] Nair J; Godschalk RW; Nair U; Owen RW; Hull WE; Bartsch H Identification of 3, *N*^4^-Etheno-5-Methyl-2′-Deoxycytidine in Human DNA: A New Modified Nucleoside Which May Perturb Genome Methylation. Chem. Res. Toxicol 2012, 25, 162–169. 22148471 10.1021/tx200392a

[58] Zhang M; Yang S; Nelakanti R; Zhao W; Liu G; Li Z; Liu X; Wu T; Xiao A; Li H Mammalian ALKBH1 Serves as an N6-MA Demethylase of Unpairing DNA. Cell Res. 2020, 30, 197–210. 32051560 10.1038/s41422-019-0237-5 PMC7054317

[59] Lee D-H; Jin S-G; Cai S; Chen Y; Pfeifer GP; O’Connor TR Repair of Methylation Damage in DNA and RNA by Mammalian AlkB Homologues. J. Biol. Chem 2005, 280, 39448–39459. 16174769 10.1074/jbc.M509881200

[60] Yang T; Cheong A; Mai X; Zou S; Woon ECY A Methylation-Switchable Conformational Probe for the Sensitive and Selective Detection of RNA Demethylase Activity. Chem. Commun 2016, 52, 6181–6184. 10.1039/c6cc01045h 27074833

[61] Chen F; Bian K; Tang Q; Fedeles BI; Singh V; Humulock ZT; Essigmann JM; Li D Oncometabolites D- and L-2-Hydroxyglutarate Inhibit the AlkB Family DNA Repair Enzymes under Physiological Conditions. Chem. Res. Toxicol 2017, 30, 1102–1110. 28269980 10.1021/acs.chemrestox.7b00009 PMC5498157

[62] Das M; Yang T; Dong J; Prasetya F; Xie Y; Wong KHQ; Cheong A; Woon ECY Multiprotein Dynamic Combinatorial Chemistry: A Strategy for the Simultaneous Discovery of Subfamily-Selective Inhibitors for Nucleic Acid Demethylases FTO and ALKBH3. Chem. Asian J 2018, 13, 2854–2867. 29917331 10.1002/asia.201800729

[63] Hu L; Lu J; Cheng J; Rao Q; Li Z; Hou H; Lou Z; Zhang L; Li W; Gong W; Structural Insight into Substrate Preference for TET-Mediated Oxidation. Nature 2015, 527, 118–122. 26524525 10.1038/nature15713

[64] Zheng G; Dahl JA; Niu Y; Fedorcsak P; Huang C-M; Li CJ; Vågbø CB; Shi Y; Wang W-L; Song S-H; ALKBH5 Is a Mammalian RNA Demethylase That Impacts RNA Metabolism and Mouse Fertility. Mol. Cell 2013, 49, 18–29. 23177736 10.1016/j.molcel.2012.10.015 PMC3646334

[65] Zhu C; Yi C Switching Demethylation Activities between AlkB Family RNA/DNA Demethylases through Exchange of Active-Site Residues. Angew. Chem. Int. Ed 2014, 53, 3659–3662. 10.1002/anie.201310050 24596302

[66] Li F; Kennedy S; Hajian T; Gibson E; Seitova A; Xu C; Arrowsmith CH; Vedadi M A Radioactivity-Based Assay for Screening Human M6A-RNA Methyltransferase, METTL3-METTL14 Complex, and Demethylase ALKBH5. SLAS Discov. 2016, 21, 290–297. 10.1177/1087057115623264 26701100

[67] Zou S; Toh JDW; Wong KHQ; Gao Y-G; Hong W; Woon ECY N6-Methyladenosine: A Conformational Marker That Regulates the Substrate Specificity of Human Demethylases FTO and ALKBH5. Sci. Rep 2016, 6, 25677. 27156733 10.1038/srep25677 PMC4860565

[68] Wang L; Song C; Wang N; Li S; Liu Q; Sun Z; Wang K; Yu S-C; Yang Q NADP Modulates RNA M6A Methylation and Adipogenesis via Enhancing FTO Activity. Nat. Chem. Biol 2020, 16, 1394–1402. 32719557 10.1038/s41589-020-0601-2

[69] Khatiwada B; Purslow JA; Underbakke ES; Venditti V N-Terminal Fusion of the N-Terminal Domain of Bacterial Enzyme I Facilitates Recombinant Expression and Purification of the Human RNA Demethylases FTO and Alkbh5. Protein Expr. Purif 2020, 167, 105540. 31740367 10.1016/j.pep.2019.105540 PMC6942671

[70] Mauer J; Luo X; Blanjoie A; Jiao X; Grozhik AV; Patil DP; Linder B; Pickering BF; Vasseur J-J; Chen Q; Reversible Methylation of M6Am in the 5′ Cap Controls MRNA Stability. Nature 2017, 541, 371–375. 28002401 10.1038/nature21022 PMC5513158

[71] Ma M; Harding HP; O’Rahilly S; Ron D; Yeo GSH Kinetic Analysis of FTO (Fat Mass and Obesity-Associated) Reveals That It Is Unlikely to Function as a Sensor for 2-Oxoglutarate. Biochem. J 2012, 444, 183–187. 22435707 10.1042/BJ20120065 PMC7617487

[72] Jia G; Yang C-G; Yang S; Jian X; Yi C; Zhou Z; He C Oxidative Demethylation of 3-Methylthymine and 3-Methyluracil in Single-Stranded DNA and RNA by Mouse and Human FTO. FEBS Lett. 2008, 582, 3313–3319. 18775698 10.1016/j.febslet.2008.08.019 PMC2577709

[73] Koivisto P; Duncan T; Lindahl T; Sedgwick B Minimal Methylated Substrate and Extended Substrate Range of *Escherichia coli* AlkB Protein, a 1-Methyladenine-DNA Dioxygenase. J. Biol. Chem 2003, 278, 44348–44354. 12944387 10.1074/jbc.M307361200

[74] Yu B; Edstrom WC; Benach J; Hamuro Y; Weber PC; Gibney BR; Hunt JF Crystal Structures of Catalytic Complexes of the Oxidative DNA/RNA Repair Enzyme AlkB. Nature 2006, 439, 879–884. 16482161 10.1038/nature04561

[75] Roy TW; Bhagwat AS Kinetic Studies of *Escherichia coli* AlkB Using a New Fluorescence-Based Assay for DNA Demethylation. Nucleic Acids Res. 2007, 35, e147. 18003660 10.1093/nar/gkm1031 PMC2175350

[76] Yu B; Hunt JF Enzymological and Structural Studies of the Mechanism of Promiscuous Substrate Recognition by the Oxidative DNA Repair Enzyme AlkB. Proc. Natl. Acad. Sci. USA 2009, 106, 14315–14320. 19706517 10.1073/pnas.0812938106 PMC2725012

[77] Ergel B; Gill ML; Brown L; Yu B; Palmer AG; Hunt JF Protein Dynamics Control the Progression and Efficiency of the Catalytic Reaction Cycle of the *Escherichia coli* DNA-Repair Enzyme AlkB. J. Biol. Chem 2014, 289, 29584–29601. 25043760 10.1074/jbc.M114.575647 PMC4207975

[78] Baldwin MR; Admiraal SJ; O’Brien PJ Transient Kinetic Analysis of Oxidative Dealkylation by the Direct Reversal DNA Repair Enzyme AlkB. J. Biol. Chem 2020, 295, 7317–7326. 32284330 10.1074/jbc.RA120.013517 PMC7247310

[79] Wang Y; Katanski CD; Watkins C; Pan JN; Dai Q; Jiang Z; Pan T A High-Throughput Screening Method for Evolving a Demethylase Enzyme with Improved and New Functionalities. Nucleic Acids Res. 2021, 49, e30. 33337498 10.1093/nar/gkaa1213 PMC7968990

[80] Karkhanina AA; Mecinović J; Musheev MU; Krylova SM; Petrov AP; Hewitson KS; Flashman E; Schofield CJ; Krylov SN Direct Analysis of Enzyme-Catalyzed DNA Demethylation. Anal. Chem 2009, 81, 5871–5875. 19518090 10.1021/ac9010556

[81] Nigam R; Anindya R *Escherichia coli* Single-Stranded DNA Binding Protein SSB Promotes AlkB-Mediated DNA Dealkylation Repair. Biochem. Biophys. Res. Commun 2018, 496, 274–279. 29326044 10.1016/j.bbrc.2018.01.043

[82] Shivange G; Kodipelli N; Anindya R 2-Hydrazinobenzothiazole-Based Etheno-Adduct Repair Protocol (HERP): A Method for Quantitative Determination of Direct Repair of Etheno-Bases. DNA Repair 2015, 28, 8–13. 25697729 10.1016/j.dnarep.2015.01.010

[83] Wei Y-F; Carter KC; Wang R-P; Shell BK Molecular Cloning and Functional Analysis of a Human CDNA Encoding an *Escherichia coli* AlkB Homolog, a Protein Involved in DNA Alkylation Damage Repair. Nucleic Acids Res. 1996, 24, 931–937. 8600462 10.1093/nar/24.5.931 PMC145711

[84] Yi C; He C DNA Repair by Reversal of DNA Damage. Cold Spring Harb. Perspect. Biol 2013, 5, a012575. 23284047 10.1101/cshperspect.a012575 PMC3579392

[85] Ougland R; Lando D; Jonson I; Dahl JA; Moen MN; Nordstrand LM; Rognes T; Lee JT; Klungland A; Kouzarides T; ALKBH1 Is a Histone H2A Dioxygenase Involved in Neural Differentiation. Stem Cells 2012, 30, 2672–2682. 22961808 10.1002/stem.1228 PMC3546389

[86] Ma C-J; Ding J-H; Ye T-T; Yuan B-F; Feng Y-Q AlkB Homologue 1 Demethylates *N*^3^-Methylcytidine in MRNA of Mammals. ACS Chem. Biol 2019, 14, 1418–1425. 31188562 10.1021/acschembio.8b01001

[87] Westbye MP; Feyzi E; Aas PA; Vågbø CB; Talstad VA; Kavli B; Hagen L; Sundheim O; Akbari M; Liabakk N-B; Human AlkB Homolog 1 Is a Mitochondrial Protein That Demethylates 3-Methylcytosine in DNA and RNA. J. Biol. Chem 2008, 283, 25046–25056. 18603530 10.1074/jbc.M803776200 PMC3259822

[88] Haag S; Sloan KE; Ranjan N; Warda AS; Kretschmer J; Blessing C; Hübner B; Seikowski J; Dennerlein S; Rehling P; NSUN3 and ABH1 Modify the Wobble Position of Mt-TRNAMet to Expand Codon Recognition in Mitochondrial Translation. EMBO J. 2016, 35, 2104–2119. 27497299 10.15252/embj.201694885 PMC5048346

[89] Kawarada L; Suzuki T; Ohira T; Hirata S; Kenjyo M; Suzuki T ALKBH1 Is an RNA Dioxygenase Responsible for Cytoplasmic and Mitochondrial TRNA Modifications. Nucleic Acids Res. 2017, 45, 7401–7415. 28472312 10.1093/nar/gkx354 PMC5499545

[90] Wu TP; Wang T; Seetin MG; Lai Y; Zhu S; Lin K; Liu Y; Byrum SD; Mackintosh SG; Zhong M; DNA Methylation on N6-Adenine in Mammalian Embryonic Stem Cells. Nature 2016, 532, 329–333. 27027282 10.1038/nature17640 PMC4977844

[91] Liu F; Clark W; Luo G; Wang X; Fu Y; Wei J; Wang X; Hao Z; Dai Q; Zheng G; ALKBH1-Mediated TRNA Demethylation Regulates Translation. Cell 2016, 167, 816–828.e16. 27745969 10.1016/j.cell.2016.09.038 PMC5119773

[92] Yang C-G; Yi C; Duguid EM; Sullivan CT; Jian X; Rice PA; He C Crystal Structures of DNA/RNA Repair Enzymes AlkB and ABH2 Bound to DsDNA. Nature 2008, 452, 961–965. 18432238 10.1038/nature06889 PMC2587245

[93] Monsen VT; Sundheim O; Aas PA; Westbye MP; Sousa MML; Slupphaug G; Krokan HE Divergent β-Hairpins Determine Double-Strand versus Single-Strand Substrate Recognition of Human AlkB-Homologues 2 and 3. Nucleic Acids Res. 2010, 38, 6447–6455. 20525795 10.1093/nar/gkq518 PMC2965238

[94] Chen B; Liu H; Sun X; Yang C-G Mechanistic Insight into the Recognition of Single-Stranded and Double-Stranded DNA Substrates by ABH2 and ABH3. Mol. BioSyst 2010, 6, 2143. 20714506 10.1039/c005148a

[95] Chen F; Tang Q; Bian K; Humulock ZT; Yang X; Jost M; Drennan CL; Essigmann JM; Li D Adaptive Response Enzyme AlkB Preferentially Repairs 1-Methylguanine and 3-Methylthymine Adducts in Double-Stranded DNA. Chem. Res. Toxicol 2016, 29, 687–693. 26919079 10.1021/acs.chemrestox.5b00522 PMC5497687

[96] Bian K; Lenz SAP; Tang Q; Chen F; Qi R; Jost M; Drennan CL; Essigmann JM; Wetmore SD; Li D DNA Repair Enzymes ALKBH2, ALKBH3, and AlkB Oxidize 5-Methylcytosine to 5-Hydroxymethylcytosine, 5-Formylcytosine and 5-Carboxylcytosine in Vitro. Nucleic Acids Res. 2019, 47, 5522–5529. 31114894 10.1093/nar/gkz395 PMC6582317

[97] You C; Wang P; Nay SL; Wang J; Dai X; O’Connor TR; Wang Y Roles of Aag, Alkbh2, and Alkbh3 in the Repair of Carboxymethylated and Ethylated Thymidine Lesions. ACS Chem. Biol 2016, 11, 1332–1338. 26930515 10.1021/acschembio.6b00085 PMC5038138

[98] Sundheim O; Vågbø CB; Bjørås M; Sousa MML; Talstad V; Aas PA; Drabløs F; Krokan HE; Tainer JA; Slupphaug G Human ABH3 Structure and Key Residues for Oxidative Demethylation to Reverse DNA/RNA Damage. EMBO J. 2006, 25, 3389–3397. 16858410 10.1038/sj.emboj.7601219 PMC1523172

[99] Chen Z; Qi M; Shen B; Luo G; Wu Y; Li J; Lu Z; Zheng Z; Dai Q; Wang H Transfer RNA Demethylase ALKBH3 Promotes Cancer Progression via Induction of TRNA-Derived Small RNAs. Nucleic Acids Res. 2019, 47, 2533–2545. 30541109 10.1093/nar/gky1250 PMC6411830

[100] Dango S; Mosammaparast N; Sowa ME; Xiong L-J; Wu F; Park K; Rubin M; Gygi S; Harper JW; Shi Y DNA Unwinding by ASCC3 Helicase Is Coupled to ALKBH3-Dependent DNA Alkylation Repair and Cancer Cell Proliferation. Mol. Cell 2011, 44, 373–384. 22055184 10.1016/j.molcel.2011.08.039 PMC3258846

[101] Brickner JR; Soll JM; Lombardi PM; Vågbø CB; Mudge MC; Oyeniran C; Rabe R; Jackson J; Sullender ME; Blazosky E; A Ubiquitin-Dependent Signalling Axis Specific for ALKBH-Mediated DNA Dealkylation Repair. Nature 2017, 551, 389–393. 29144457 10.1038/nature24484 PMC6458054

[102] Meyer KD; Saletore Y; Zumbo P; Elemento O; Mason CE; Jaffrey SR Comprehensive Analysis of MRNA Methylation Reveals Enrichment in 3′ UTRs and near Stop Codons. Cell 2012, 149, 1635–1646. 22608085 10.1016/j.cell.2012.05.003 PMC3383396

[103] Dominissini D; Moshitch-Moshkovitz S; Schwartz S; Salmon-Divon M; Ungar L; Osenberg S; Cesarkas K; Jacob-Hirsch J; Amariglio N; Kupiec M; Topology of the Human and Mouse M6A RNA Methylomes Revealed by M6A-Seq. Nature 2012, 485, 201–206. 22575960 10.1038/nature11112

[104] Ensfelder TT; Kurz MQ; Iwan K; Geiger S; Matheisl S; Müller M; Beckmann R; Carell T ALKBH5-Induced Demethylation of Mono- and Dimethylated Adenosine. Chem. Commun 2018, 54, 8591–8593. 10.1039/c8cc03980a 30010678

[105] Takahashi H; Hase H; Yoshida T; Tashiro J; Hirade Y; Kitae K; Tsujikawa K Discovery of Two Novel ALKBH5 Selective Inhibitors That Exhibit Uncompetitive or Competitive Type and Suppress the Growth Activity of Glioblastoma Multiforme. Chem. Biol. Drug Des 2022, 100, 1–12. 35384315 10.1111/cbdd.14051

[106] Frayling TM; Timpson NJ; Weedon MN; Zeggini E; Freathy RM; Lindgren CM; Perry JRB; Elliott KS; Lango H; Rayner NW; A Common Variant in the *FTO* Gene Is Associated with Body Mass Index and Predisposes to Childhood and Adult Obesity. Science 2007, 316, 889–894. 17434869 10.1126/science.1141634 PMC2646098

[107] Gerken T; Girard CA; Tung Y-CL; Webby CJ; Saudek V; Hewitson KS; Yeo GSH; McDonough MA; Cunliffe S; McNeill LA; The Obesity-Associated *FTO* Gene Encodes a 2-Oxoglutarate-Dependent Nucleic Acid Demethylase. Science 2007, 318, 1469–1472. 17991826 10.1126/science.1151710 PMC2668859

[108] Wei J; Liu F; Lu Z; Fei Q; Ai Y; He PC; Shi H; Cui X; Su R; Klungland A; Differential M6A, M6Am, and M1A Demethylation Mediated by FTO in the Cell Nucleus and Cytoplasm. Mol. Cell 2018, 71, 973–985.e5. 30197295 10.1016/j.molcel.2018.08.011 PMC6151148

[109] Fu Y; Jia G; Pang X; Wang RN; Wang X; Li CJ; Smemo S; Dai Q; Bailey KA; Nobrega MA; FTO-Mediated Formation of N6-Hydroxymethyladenosine and N6-Formyladenosine in Mammalian RNA. Nat. Commun 2013, 4, 1798. 23653210 10.1038/ncomms2822 PMC3658177

[110] Chen W; Zhang L; Zheng G; Fu Y; Ji Q; Liu F; Chen H; He C Crystal Structure of the RNA Demethylase ALKBH5 from Zebrafish. FEBS Lett. 2014, 588, 892–898. 24561204 10.1016/j.febslet.2014.02.021 PMC3982313

[111] Waheed SO; Ramanan R; Chaturvedi SS; Ainsley J; Evison M; Ames JM; Schofield CJ; Christov CZ; Karabencheva-Christova TG Conformational Flexibility Influences Structure–Function Relationships in Nucleic Acid N-Methyl Demethylases. Org. Biomol. Chem 2019, 17, 2223–2231. 30720838 10.1039/c9ob00162j

[112] Wang B; Cao Z; Sharon DA; Shaik S Computations Reveal a Rich Mechanistic Variation of Demethylation of N-Methylated DNA/RNA Nucleotides by FTO. ACS Catal. 2015, 5, 7077–7090.

[113] Adams JM; Cory S Modified Nucleosides and Bizarre 5′-Termini in Mouse Myeloma MRNA. Nature 1975, 255, 28–33. 1128665 10.1038/255028a0

[114] Wei C-M; Gershowitz A; Moss B N6, O2′-Dimethyladenosine a Novel Methylated Ribonucleoside next to the 5′ Terminal of Animal Cell and Virus MRNAs. Nature 1975, 257, 251–253. 1161029 10.1038/257251a0

[115] Cliffe LJ; Siegel TN; Marshall M; Cross GAM; Sabatini R Two Thymidine Hydroxylases Differentially Regulate the Formation of Glucosylated DNA at Regions Flanking Polymerase II Polycistronic Transcription Units throughout the Genome of Trypanosoma Brucei. Nucleic Acids Res. 2010, 38, 3923–3935. 20215442 10.1093/nar/gkq146 PMC2896530

[116] Vainio S; Genest P-A; ter Riet B; van Luenen H; Borst P Evidence That J-Binding Protein 2 Is a Thymidine Hydroxylase Catalyzing the First Step in the Biosynthesis of DNA Base J. Mol. Biochem. Parasitol 2009, 164, 157–161. 19114062 10.1016/j.molbiopara.2008.12.001

[117] van Leeuwen F; Taylor MC; Mondragon A; Moreau H; Gibson W; Kieft R; Borst P β-D-Glucosyl-Hydroxymethyluracil Is a Conserved DNA Modification in Kinetoplastid Protozoans and Is Abundant in Their Telomeres. Proc. Natl. Acad. Sci. USA 1998, 95, 2366–2371. 9482891 10.1073/pnas.95.5.2366 PMC19348

[118] Toaldo CB; Kieft R; Dirks-Mulder A; Sabatini R; van Luenen HGAM; Borst P A Minor Fraction of Base J in Kinetoplastid Nuclear DNA Is Bound by the J-Binding Protein 1. Mol. Biochem. Parasitol 2005, 143, 111–115. 15935489 10.1016/j.molbiopara.2005.05.001 PMC10278234

[119] Torabifard H; Cisneros GA Insight into Wild-Type and T1372E TET2-Mediated 5hmC Oxidation Using Ab Initio QM/MM Calculations. Chem. Sci 2018, 9, 8433–8445. 30542593 10.1039/c8sc02961j PMC6244454

[120] Waheed SO; Chaturvedi SS; Karabencheva-Christova TG; Christov CZ Catalytic Mechanism of Human Ten-Eleven Translocation-2 (TET2) Enzyme: Effects of Conformational Changes, Electric Field, and Mutations. ACS Catal. 2021, 11, 3877–3890.

[121] Waheed SO; Varghese A; Chaturvedi SS; Karabencheva-Christova TG; Christov CZ How Human TET2 Enzyme Catalyzes the Oxidation of Unnatural Cytosine Modifications in Double-Stranded DNA. ACS Catal. 2022, 12, 5327–5344. 36339349 10.1021/acscatal.2c00024 PMC9629818

[122] Liu MY; Torabifard H; Crawford DJ; DeNizio JE; Cao X-J; Garcia BA; Cisneros GA; Kohli RM Mutations along a TET2 Active Site Scaffold Stall Oxidation at 5-Hydroxymethylcytosine. Nat. Chem. Biol 2017, 13, 181–187. 27918559 10.1038/nchembio.2250 PMC5370579

[123] Kataoka H; Yamamoto Y; Sekiguchi M A New Gene (AlkB) of *Escherichia coli* That Controls Sensitivity to Methyl Methane Sulfonate. J. Bacteriol 1983, 153, 1301–1307. 6337994 10.1128/jb.153.3.1301-1307.1983 PMC221777

[124] Wang J; Qi R; Li H; Christov C; Lehnert N; Li D Genetic and Epigenetic Biomarkers Related to 2-Oxoglutarate/Fe(II)-Dependent Oxygenases and Implications for Disease and Toxicology. In Biomarkers in Toxicology; Patel VB, Preedy VR, Rajendram R, Eds.; Biomarkers in Disease: Methods, Discoveries and Applications; Springer International Publishing: Cham, Switzerland, 2022; pp. 1–28.

[125] Fang D; Cisneros GA Alternative Pathway for the Reaction Catalyzed by DNA Dealkylase AlkB from Ab Initio QM/MM Calculations. J. Chem. Theory Comput 2014, 10, 5136–5148. 25400523 10.1021/ct500572t PMC4230374

[126] Quesne MG; Latifi R; Gonzalez-Ovalle LE; Kumar D; de Visser SP Quantum Mechanics/Molecular Mechanics Study on the Oxygen Binding and Substrate Hydroxylation Step in AlkB Repair Enzymes. Chem.–A Eur. J 2014, 20, 435–446. 10.1002/chem.201303282 PMC3994944 24339041

[127] Fang D; Lord RL; Cisneros GA Ab Initio QM/MM Calculations Show an Intersystem Crossing in the Hydrogen Abstraction Step in Dealkylation Catalyzed by AlkB. J. Phys. Chem. B 2013, 117, 6410–6420. 23642148 10.1021/jp403116e

[128] Wang B; Usharani D; Li C; Shaik S Theory Uncovers an Unusual Mechanism of DNA Repair of a Lesioned Adenine by AlkB Enzymes. J. Am. Chem. Soc 2014, 136, 13895–13901. 25203306 10.1021/ja507934g

[129] Dai Q; Zheng G; Schwartz MH; Clark WC; Pan T Selective Enzymatic Demethylation of N^2^,N^2^-Dimethylguanosine in RNA and Its Application in High-Throughput TRNA Sequencing. Angew. Chem. Int. Ed 2017, 56, 5017–5020. 10.1002/anie.201700537 PMC5497677 28371071

[130] Li M-M; Nilsen A; Shi Y; Fusser M; Ding Y-H; Fu Y; Liu B; Niu Y; Wu Y-S; Huang C-M; ALKBH4-Dependent Demethylation of Actin Regulates Actomyosin Dynamics. Nat. Commun 2013, 4, 1832. 23673617 10.1038/ncomms2863 PMC3674258

[131] Mielecki D; Zugaj DŁ; Muszewska A; Piwowarski J; Chojnacka A; Mielecki M; Nieminuszczy J; Grynberg M; Grzesiuk E Novel AlkB Dioxygenases—Alternative Models for In Silico and In Vivo Studies. PLoS ONE 2012, 7, e30588. 22291995 10.1371/journal.pone.0030588 PMC3265494

[132] Tsujikawa K; Koike K; Kitae K; Shinkawa A; Arima H; Suzuki T; Tsuchiya M; Makino Y; Furukawa T; Konishi N; Expression and Sub-Cellular Localization of Human ABH Family Molecules. J. Cell. Mol. Med 2007, 11, 1105–1116. 17979886 10.1111/j.1582-4934.2007.00094.x PMC4401260

[133] Zhang L-S; Xiong Q-P; Peña Perez S; Liu C; Wei J; Le C; Zhang L; Harada BT; Dai Q; Feng X; ALKBH7-Mediated Demethylation Regulates Mitochondrial Polycistronic RNA Processing. Nat Cell Biol 2021, 23, 684–691. 34253897 10.1038/s41556-021-00709-7 PMC8716185

[134] Bobiak ML Biochemical Characterization of Human AlkBH7. Doctoral Dissertation, Stony Brook University, Stony Brook, NY, USA, 2009.

[135] Pastore C; Topalidou I; Forouhar F; Yan AC; Levy M; Hunt JF Crystal Structure and RNA Binding Properties of the RNA Recognition Motif (RRM) and AlkB Domains in Human AlkB Homolog 8 (ABH8), an Enzyme Catalyzing TRNA Hypermodification. J. Biol. Chem 2012, 287, 2130–2143. 22065580 10.1074/jbc.M111.286187 PMC3265892

[136] Tang Q; Cai A; Bian K; Chen F; Delaney JC; Adusumalli S; Bach AC; Akhlaghi F; Cho BP; Li D Characterization of Byproducts from Chemical Syntheses of Oligonucleotides Containing 1-Methyladenine and 3-Methylcytosine. ACS Omega 2017, 2, 8205–8212. 29214236 10.1021/acsomega.7b01482 PMC5709782

